# Characterization of a distinct form of vimentin in the neurodegenerative brain

**DOI:** 10.1186/s40478-026-02324-9

**Published:** 2026-05-22

**Authors:** Abdulkhalek Dakhel, Johanna Vestin, Vilmantas Giedraitis, Dag Nyholm, Martin Ingelsson, Anna Erlandsson

**Affiliations:** 1 Department of Public Health and Caring Sciences, Molecular Geriatrics, Uppsala University, Uppsala, Sweden; 2 Department of Medical Sciences, Neurology, Uppsala University, Uppsala, Sweden; 3 University Health Network, Krembil Brain Institute, Toronto, ON Canada; 4 Tanz Centre for Research in Neurodegenerative Diseases, Departments of Medicine and Laboratory Medicine and Pathobiology, University of Toronto, Toronto, ON Canada

**Keywords:** Vimentin, Astrocytes, Alzheimer's disease, Parkinson’s disease, Amyloid-beta, Alpha-synuclein

## Abstract

Alzheimer’s disease (AD) and Parkinson’s disease (PD) are characterized by brain accumulation of aggregated proteins, leading to neuronal and glial dysfunction. Compelling data indicate that changes in vimentin expression, structure, and localization reflect alterations in cellular homeostasis and may correlate with disease progression. Yet, the involvement of abnormal vimentin in AD and PD remains unclear. Here, we have thoroughly characterized the distribution of modified, disease-associated vimentin in the AD and PD brain parenchyma, as well as in cerebrospinal fluid (CSF), hiPSC-derived astrocytes and organoids. For this purpose, we used the form-specific vimentin antibody [84-1], originally generated to recognize abnormal vimentin on the surface of sarcoma cells. The 84-1 vimentin reactivity pattern was characterized through immunohistochemistry and proximity ligation assay. Moreover, proteomic analysis, Western blot, and ELISA were performed to quantify 84-1 vimentin levels and gain insights into its molecular features. Our data demonstrate that 84-1 can identify a distinct population of vimentin in the human brain that shows low affinity to commonly used vimentin antibodies. The 84-1 vimentin is enriched in disease conditions and forms distinct deposits in the affected regions of the AD and PD brain, often colocalizing with pathological protein aggregates. Interestingly, the 84-1 vimentin pool consists mainly of cleaved proteoforms and reduction-resistant aggregates. Moreover, 84-1 vimentin levels are elevated in the CSF of AD and PD patients as well as in the culture medium of human astrocytes exposed to αSyn or Aβ fibrils. Taken together, our data highlights the importance of modified vimentin in neurodegeneration and presents 84-1 vimentin as a potential biomarker and future treatment target for AD and PD.

**Supplementary Information:**

The online version contains supplementary material available at 10.1186/s40478-026-02324-9.

## Introduction

Alzheimer’s disease (AD) and Parkinson’s disease (PD) are the two most prevalent neurodegenerative disorders, each characterized by distinct pathological features and clinical manifestations. AD symptoms are primarily associated with hippocampal dysfunction, resulting in cognitive decline and memory loss [18]. Parkinson’s disease, on the other hand, is characterized by motor dysfunction, including bradykinesia, tremors, and rigidity, caused by the loss of dopaminergic innervation in the substantia nigra and dorsal striatum [5]. A major hallmark of both diseases is the buildup of aggregated proteins in the brain. In AD, amyloid-beta (Aβ) accumulates as plaques, while hyperphosphorylated tau forms neurofibrillary tangles [42], and in PD, alpha-synuclein (αSyn) aggregates into Lewy bodies and Lewy neurites [5].

Growing evidence indicates that neuroinflammation, including astrocyte activation, plays a central role in AD and PD progression. Astrocytes are known to act as hubs for neural networks, connecting and influencing neurons, brain vasculature, and other brain cells. Their physiological functions include metabolic support, neurotransmitter homeostasis, and blood-brain barrier maintenance. In the diseased brain, astrocytes are converted to a reactive state, resulting in an increased capacity to ingest aggregated proteins and cellular debris [4, 38]. Moreover, reactive astrocytes release proinflammatory mediators, which could aggravate the immune reaction [8]. Importantly, chronic astrocyte activation compromises the cells’ ability to fulfil physiological tasks, which may significantly worsen the neurodegenerative disease course [28, 30]. On the other hand, the astrocyte-immune cell interplay is essential for recruitment of phagocytic cells, which protect the brain from damage by clearing pathogens [21]. Hence, the astrocytic response is complex and entails both protective and harmful effects, requiring careful characterization.

Disease-associated astrocytes are identified by their elevated levels of reactivity markers, including vimentin [43]. Vimentin is a widely expressed intermediate filament protein that is crucial for maintaining cell integrity and motility. Moreover, vimentin is highly involved in cell-to-cell communication, organelle movement, and inflammation [11]. In the AD brain, astrocytes surrounding amyloid plaques have substantially increased vimentin expression [19], and in PD, there is a close connection between vimentin-rich aggresomes and Lewy bodies [24, 31]. These observations indicate a central role of vimentin in AD/PD pathology, but the exact mechanisms remain unclear.

The diverse functions of vimentin are largely dependent on its dynamic structure, achieved by post-translational modifications (PTMs). For example, phosphorylation at specific subunits regulates cell signaling and migration [52] by affecting vimentin filament assembly [16]. In neurodegenerative diseases, aberrant vimentin PTMs may contribute to pathological astrocyte-mediated changes. It has been shown that hyperphosphorylation of vimentin, due to diphenyl treatment, increases astrocyte reactivity and consequently worsens neuronal damage [14], which may explain the induction of PD in exposed individuals [50]. Despite these indications, vimentin and its modifications have been mainly studied in the periphery, while its expression and processing in the brain remain poorly understood. Here, we aimed to map the distribution and expression of vimentin using a form-specific vimentin antibody clone 84-1 that has not been characterized in the human brain before. Our data show that 84-1 identifies previously unresolved vimentin variants that are accumulated in the brain and CSF of AD and PD patients.

## Materials and methods

### Study samples

The Regional Ethical Review Board in Uppsala, Sweden, and the Swedish Ethical Review Authority had approved all parts of the study.

#### Brain tissue sections

Paraffin-embedded brain samples were provided by Uppsala Biobank (ethical number 2012/494). Cases were chosen based on strict selection criteria. The subjects were within the age range of 70 to 79 years and the *post-mortem* time did not exceed 144 h. Individuals with AD (*n* = 4) had dementia and displayed a high level of Alzheimer’s disease neuropathologic changes (ADNC). For PD individuals (*n* = 4), αSyn pathology was substantial, i.e. Braak stage ≥ 4. Control individuals for AD (*n* = 4) and for PD (*n* = 4) were age-matched and displayed no glial hyperphosphorylated tau pathology, i.e. no ARTAG and no Aβ, αSyn or TDP43 pathology, and neuronal hyperphosphorylated tau pathology was limited to locus coeruleus. A summary of basic donor characteristics is presented in Supplementary Table S1.

#### CSF samples

All CSF samples were obtained from the Uppsala Biobank (ethical permit numbers 2013/187 and 2020/02390). The CSF samples from AD individuals (*n* = 7) were collected from early-disease patients at the time of diagnosis. The Average Mini-Mental State Examination (0–30) score of AD and control donors is reported in Supplementary Table S2. CSF from PD individuals (*n* = 7) was sampled on average 10 years (± 5.6) after diagnosis. Age-matched control samples for AD (*n* = 7) and for PD (*n* = 7) were also collected. Written informed consent was provided by all participants or by their direct next-of-kin. A summary of basic donor characteristics is presented in Supplementary Table S2.

### Deparaffinization

Five-micrometer paraffin-embedded sections from either PD patients (coronal striatum), AD patients (hippocampus), or age-matched controls were used in the analysis. Prior to labeling with immunohistochemistry or proximity ligation assay, the sections were deparaffinized in xylene 2 × 10 min, rehydrated in 99.5% ethanol for 20 min, 95% ethanol for 10 min and 70% ethanol for 10 min, followed by a washing step in PBS.

### Immunohistochemistry (IHC)

#### Fluorescent microscopy

Blocking and permeabilization were performed with 5% NGS (Bionordika, S-1000) and 0.1% saponin (S7900-25G, Merk) in PBS for 1 h on shake at room temperature (RT). Primary antibodies (Table 1) were diluted in 0.5% NGS and 0.1% Saponin in PBS and incubated with the sections on shake at 4 °C overnight (ON). The following day, the sections were washed in PBS 3 × 5 min. Fluorescent Alexa 488, 555 or 647 goat secondary antibodies (1:200 Thermo Fisher Scientific) were diluted in 0.5% NGS and 0.1% Saponin in PBS and added to the sections for 1 h at 37 °C, followed by 3 × 5 min wash in PBS. To quench tissue autofluorescence, the tissue slides were dipped in 70% ethanol and incubated in 0.3% Sudan black for 20 min, followed by washing in 70% ethanol for 5 min and PBS for 5 min, before mounting in EverBrite Hardset mounting medium with DAPI (23004, VWR). Fluorescence images were captured at 40x dry and 63x oil magnification, using the Leica DMi8 microscope. 3D volumetric reconstructions were made from 63x z-stacks using the Volume Viewer plugin in Fiji.

#### 84-1 immunoreactivity characterization

84-1 staining pattern was analyzed against immunoreactivity of S100b, GFAP or reference (Ref.) vimentin. Each staining pair was analyzed in brain sections from five different control individuals. Hippocampus and striatal sections were taken from each individual. To define the regions of interest, the DG, CA3-CA1 was outlined on the hippocampus slides, while the caudate nucleus and putamen were outlined on the striatal section, and full tile scans of the outlined regions were captured at 40x. Fiji was then used to analyze the ratio of overlapping positive area in relation to the total area for each marker in the pair.

#### DAB staining

Brain tissue sections were subjected to 3,3′-diaminobenzidine (DAB) staining following the manufacturer’s protocol (Agilent, K346711-2). Briefly, sections were incubated with either Ref. Vim or the 84-1 Vim primary antibody (Table 1), followed by an HRP-tagged secondary antibody. DAB substrate was applied until the signal became visible, and the sections were then washed with deionized water and mounted using EverBrite Hardset mounting medium with DAPI (23004, VWR). To confirm signal specificity, an absorption control was performed by pre-incubating the 84-1 antibody with a ten-fold molar excess of recombinant vimentin (Fisher Scientific, 17810393) at 4º C ON prior to staining. All sections were analyzed under identical conditions, and images were captured at 20x or 40x magnification, using a Leica K3C camera on the Leica DMi8 microscope.

### Culturing of human iPSC-derived cortical organoids

Human cortical organoids were generated from induced pluripotent stem cells (iPSC, CNTRL9 II cell line, RRID: CVCL_JL74). The hiPS cells were cultured on 6-well cell-culture plates (Thermo, 140675) coated with 50 µg/mL vitronectin (Thermo, A14700), using E8 + medium (Thermo, A1517001). The medium was replaced every day, and the cells were passaged at 70–80% confluency, using Accutase (Thermo, 11599686). The cells were resuspended in E8 + medium fortified with 1x Y27 (Cayman, Caym10005583-10) and transferred as single cells to an AggreWell (Stemcell, 34411) (3 M cells/well). The AggreWell plate was centrifuged at 300 g for 5 min and placed in an incubator over night to create spheroids. The spheroids were then gently collected and sieved through a cell strainer (Fisher Scientific, 10737821). The spheroids were kept on UltraLow adhesion 100 mm plates (Thermo, 16855831) in E6 + medium (Thermo, A1516401) fortified with 10 µM SB431542 (Cayman, Caym13031-10), 0,25 µM XAV939 (Cayman, Caym13596-10) and 2.5 µM Dorsomorphine hydrochloride (Cayman, Caym21207-25). From the second day, the medium was changed every day. After 5 days the medium was substituted for Neurobasal (Thermo, 21103049), containing 1% penicillin-streptomycin (Thermo Fisher, 11548876), 1x B27-noVitA (Fisher Scientific, 11500446) and 1x GlutaMAX (Thermo Fisher, 35050038). For the first two weeks in Neurobasal, the medium was replaced every day, but for the coming four weeks it was replaced every other. From day 6–24, 27 ng/mL FGF2 (Fisher Scientific, 10222253) and 20 ng/mL EGF (Fisher Scientific, 17159651) were added to the medium. From day 25–43, 20 ng/mL NT-3 (Fisher Scientific, 17109511) and 20 ng/mL BDNF (Fisher Scientific, 17169321) were added to the medium. From day 44 and onwards, the medium had no additional factors and it was changed twice a week. Organoids were cultured for a total of 37 weeks then were fixed overnight in 4% PFA. The next morning, they were transferred to 30% sucrose solution until sectioning. Before sectioning, organoids were snap-frozen on dry ice and then sectioned as 12 μm thick slices using a Leica CM1860 UV cryostat and placed on coverslips for immunostaining.

### In-situ Proximity ligation assay

Proximity ligation assay (PLA) was performed using the NaveniBright - MR, HRP kit (Navinci Diagnostics, NB.MR.HRP.100) following the manufactures protocol. After deparaffinization, antigen retrieval was carried out by boiling the sections in 25mM citric buffer, pH 6.0 for 2 min, followed by incubation in 3% hydrogen peroxide for 30 min at RT for peroxidase quenching. A mouse primary antibody against vimentin (clone 84-1) and a rabbit antibody against phosphorylated S129 α-synuclein (p- αsyn S129) (Table 1) were used to develop the PLA signal. Slides were analyzed under a Zeiss Axio Observer Z1 and the Zeiss Axiocam 503 Color with the Zen 2.0 Software (Zeiss). Pictures were captured using a 40x or 63x oil objective.

### Differentiation and culture of human iPSC-derived astrocytes

Human astrocytes were generated from neuroepithelial-like stem (NES) cells, produced from human iPSCs (Cntrl9 II cell line, RRID: CVCL_JL74), according to a well-established protocol with minor modifications [13, 29]. For differentiation to astrocytes, NES cells were plated at 4000 cells/cm^2^ on 20 µg/ml poly-L-ornithine hydrobromide (#P3655, Merck, Darmstadt, Germany) and 4 µg/ml Laminin2020 (#L2020, Merck) double-coated culture vessels. Astrocyte differentiation medium consisted of advanced DMEM/F12 (#11540446) supplemented with 1% penicillin–streptomycin (#11548876), 2% B27 (#11530536), 1% non-essential amino acids (#11140050), and 1% L-glutamine (#25030-024) (all from Thermo Fisher Scientific, MA, USA). The following factors were added fresh to the medium just before use: recombinant human fibroblast growth factor-basic (bFGF) 8 ug/ml (#11390832, Thermo Fisher Scientific), heregulin 10 ng/ml (#SRP3055, Merck), activin A 10 ng/ml (#120-14E, PeproTech, NJ, USA), insulin-like growth factor 1 (IGF-1) 200 ng/ml (#SRP3069, Merck) and human ciliary neurotropic factor (CNTF) 20 ng/ml (#PHC7015, Thermo Fisher Scientific). The medium was changed every other day and astrocytes were passaged using Trypsin-EDTA (#15400054, Thermo Fisher Scientific), followed by 1:1 wash with 10% heat inactivated FBS in PBS at 80% confluency. For experiments, the astrocytes were seeded at a concentration of 5000 cells/cm2 on double coated plates.

### Protein aggregates production and treatment

#### Production and labelling of αSyn fibrils

Alpha-synuclein preformed fibrils (αSyn) were generated using endotoxin-free monomeric αSyn (AnaSpec, A5555-1000) [36]. Shortly, the monomers were dissolved in PBS at a concentration of 5 mg/ml and left on a shaker at 1000 rpm for 7d. Afterwards, αSyn PFF were diluted to a working concentration of 2 mg/mL in PBS and stored at − 80 °C until use. A fraction of the fibrils was fluorescently labelled using Cy3AM (GE Healthcare, PA33000) Protein labeling kit.

#### Production of Aβ fibrils

Fluorescent HiLyte™ Fluor 555 labelled human Aβ_42_ monomers (#60480-01, AnaSpec) or unlabeled Aβ_42_ monomers (SP-BA42-1, Innovagen) were aggregated into insoluble fibrils according to a well-established protocol [37]. The synthetic Aβ_42_ peptides were incubated in 10 mM NaOH and 10x PBS (#11530486, Thermo Fisher Scientific) to a concentration of 2 mg/ml and left on a shaker at 1500 rpm at 37 °C for 4 days. Fibrils were then obtained through centrifugation and diluted in peptide PBS to a final concentration of 0.5 mg/ml and stored at -80 °C until used.

#### Production of amyloid β soluble aggregates

Aβ_42_ soluble aggregates were produced according to a well-established protocol [12, 41] In short, synthetic Aβ_42_ peptides (Innovagen, SP-BA42-1) were dissolved in 10 mM NaOH, mixed with phosphate-buffered saline (PBS) to a concentration of 443 µM (2 mg/ml) and incubated at 37 °C for 30 min. The samples were centrifuged for 5 min at 17,900 g at 4 °C to remove any insoluble aggregates, followed by 1:4 dilution of supernatant in sterile PBS to a final concentration of 0.5 mg/ml. The protofibril-specific ELISA, based on mAb158 [41], was used to confirm the Aβ_42_ soluble aggregate concentration and transmission electron microscopy (TEM) was used to verify their shape.

#### Cell exposure

Prior to each experiment, the required volume of αsyn or Aβ stock fibrils was diluted to a volume of 150 µl in PBS to be sonicated for 1 min, 1s off/1s on at an amplitude of 20% (#VCX130, Vi-bra Cell sonicator, Sonics). The sonicated mixture was then mixed in culture media to a final working concentration of 0.5 µM for αsyn and 0.2 µM for Aβ. Astrocytes were incubated with the fibrils for three days, while control cultures received aggregates-free medium. At day 3 the cells were thoroughly washed and then cultured further in aggregates-free medium for another 4 days (3 + 4d). For western blot analysis, cells were treated with unlabeled fibrils, while fluorescent fibrils were used for cells that were fixed and stained.

#### Organoids exposure

6-month-old organoids were transferred to individual wells of a 96-well UltraLow adhesion plate (Fisher Scientific, 174932) containing 100 µL of medium. Then, another 100 µL of medium was added containing 0.2 µM Aβ_42_ soluble aggregates. The organoids were cultured separately, and the medium was replaced every other day. After one week, the organoids were transferred to culture dishes and cultured for an additional period of 12 weeks with regular medium changes (organoids were 37 weeks old at the time of analysis). For imaging organoids were fixed overnight in 4% PFA. The next day, they were transferred to 30% sucrose solution until sectioning. Organoids were snap-frozen on dry ice and sectioned as 10 μm thick slices using a Leica CM1860 UV cryostat and placed on coverslips to dry and immunohistochemistry staining was performed as described above.

### Immunocytochemistry (ICC)

Cultures were fixed in 4% paraformaldehyde (PFA) (#P6148, Merck) in PBS and then washed twice with PBS. Blocking and permeabilization were performed with 5% normal goat serum (NGS) (Bionordika, S-1000) and 0.1% saponin in PBS for 30 min at RT. Primary antibodies (Table 1) were diluted in 0.5% NGS and 0.1% saponin in PBS and added to the cultures for 2 h at RT. Thereafter, cells were washed 3 times with PBS and incubated with Alexa 488, 555 or 647 goat secondary antibodies (1:200, Thermo Fisher Scientific) for 1 h at 37 °C. After additional washes, the cells were mounted, using Ever Brite Hardset Mounting medium with DAPI. Fluorescence images were captured at 40x dry and 63x oil magnification, using the Leica DMi8 microscope.

### Immunoprecipitation of vimentin

100 mg frozen cortical human brain tissue was dissected in 500 µl lysis buffer fortified with protease/phosphatase inhibitor cocktail using a scalpel. The mixture was then homogenized in a Precellys Evolution (Bertin Technologies, France) and the homogenate was left on ice for 30 min with occasional vortexing to extract soluble proteins. Then, the mixture was centrifuged at 16,000 g for 1 h. The supernatant (soluble fraction) was removed and the pellet (insoluble fraction) was resuspended in the same volume of 1% SDS and sonicated for 1 min with 2 s pulse on/ 1 s pulse off at an amplitude of 25%. Samples were kept in -70 °C until analysis. To isolate vimentin, a Dynabeads™ Co-Immunoprecipitation Kit (FisherScientific,10365438) was used, according to the manufacturer’s instructions. 84-1 was used to coat the magnetic beads overnight. 100 µl of the brain soluble/insoluble fraction was boiled in 1mM DTT for 10 min and then incubated with the coated beads for 30 min at 4 °C followed by washing steps and finally elution of the bound proteins.

### Immunoblotting

#### Cell lysis and media collection

Culture medium was carefully aspirated and frozen directly at -70 until analysis. For cell lysates, 150 µl of ice-cold lysis buffer (20 mM Tris pH 7.5, 0.5% Triton X-100, 0.5% deoxycholic acid, 150 mM NaCl, 10 mM EDTA, 30 mM NaPyro) supplemented with Halt™ Protease and Phosphatase Inhibitor Cocktail (Thermo Fisher Scientific, #78442) was added onto the cells. The cell lysates were transferred to protein LoBind tubes (Eppendorf, #0030108094) and incubated for 30 min on ice prior to centrifugation at 12,000 g for 15 min at 4 °C. The supernatant containing the insoluble fraction was separated from the pellet (insoluble fraction) into separate tubes. The insoluble fraction was resuspended in 1% SDS in PBS and sonicated for 1 min with 2 s pulse on/ 1 s pulse off at an amplitude of 25%. All samples were kept in -70 °C until analysis.

#### Western blot analysis

Protein concentration in cell lysates was measured using the Pierce BCA protein kit (Thermo Fisher Scientific, #23225), according to the manufacturer’s instructions. Equal protein amounts of lysates and equal volumes of media were mixed with Bolt LDS Sample buffer (Thermo Fisher Scientific, 13276499) and Sample Reducing agent (Thermo Fisher Scientific, B0009) and incubated for 5 min at 95 °C to denature the proteins. Samples were then loaded on a Bolt 4–12% Bis–Tris plus gel and run in Bolt MES sodium dodecyl sulfate (SDS) running buffer (Thermo Fisher Scientific, 13266499) for 25 min at 200 V. PageRuler™ Plus Prestained Protein Ladder, 10 to 250 kDa (Thermo Fisher Scientific, 26619,) was included to determine protein size. Transfer to a PVDF membrane was performed using semi-dry transfer blot at the mixed-range settings for molecular weight (Power blotter, Invitrogen). Blocking of the membrane was performed in 5% Blotting-Grade Blocker (VWR, 22012) in 0.1% tris-buffered saline-Tween (TBS-T) for 1 h on shake at RT, prior to ON incubation with primary antibodies (Table 1) at 4 °C on shake. Following 20-minute washes in TBS-T, the membrane was incubated with HRP conjugated secondary antibodies (1:10000, Thermo Fisher Scientific) in 5% Blotting-Grade Blocker in 0.1% TBS-T for 1 h on shake at RT. Development of the membrane was performed with ECL (GE Healthcare, IL, USA) using a ChemiDoc XRS with Image Lab Software to visualize the immunoreactive bands (Bio-Rad Laboratories).

#### Western blot quantification

The intensity of the detected immunoreactive bands was measured using the Image Lab software. Each band was normalized to the total protein of that respective lane, using the No-Stain™ protein labeling reagent (Thermo Fisher Scientific, A44717). After the proteins were transferred to the PVDF membrane, the membrane was washed in ultrapure water and incubated for 10 min in No-Stain™ protein labeling solution. Following a second washing step, the membranes were imaged using a ChemiDoc XRS and the acquired images were used for normalization of each immunoreactive band, detected after immunoblotting. The intensity values were taken directly from the Image Lab software.

### Mass spectrometry

The immunoprecipitation elute from 100 mg frozen cortical human brain tissue was prepared for LC-MS/MS analysis. Shortly, the proteins were reduced, alkylated, on-filter digested by trypsin using 3 kDa centrifugal spin filter (Millipore, Ireland). The collected peptide filtrate was vacuum centrifuged to dryness using a SpeedVac system. The sample was dissolved in 30 µL 0.1% formic acid and further diluted 3 times prior to LC-MS/MS analysis. The resulting peptides were separated in reversed-phase on a C18-column and electrosprayed on-line to a QEx-Orbitrap mass spectrometer (Thermo Finnigan) with 90 min gradient. Tandem mass spectrometry was performed applying HCD. Database searching was performed using FragPipe (Nesvilab). MSfragger cleavage parameters were set run on Enzymatic and SEMI with Trypsin. Fixed modification was Carbamidomethyl (C), and variable modifications were Oxidation (M), N-terminal acetylation ([^), and phosphorylation (STY). The proteomic output was cross-referenced with the cRAP protein database to determine and exclude common contaminants.

### Measurement of vimentin in CSF

Vimentin concentration in CSF samples was measured using an in-house sandwich ELISA assay. First, 96-well half-area plates were coated with 0.75 µg/ml 84-1 overnight at 4 °C. The plate was then blocked with 1% BSA in PBS for 2 h at RT. CSF samples were first boiled in 1mM DTT at 95 °C for 10 min. Recombinant human vimentin (FisherScientific, 17810393) was used as a standard. Following overnight incubation at 4 °C, the plate was washed. Ref. vimentin antibody (Table 1) (0.5 µg/ml) was used as primary antibody and HRP-conjugated anti-chicken (Thermofisher, PA1-28798) (1:2000) antibody was used for detection. All dilutions were made in ELISA incubation buffer (0.1% BSA and 0.05% Tween-20 in PBS). Signals were developed using K blue aqueous TMB substrate (Neogen, 331,177), stopped with 1 M H2SO4, and read with a spectrophotometer at 450 nm. Interpolation of sample concentrations was performed using GraphPad Prism v10 and the hyperbola as a model.

### Statistical analyses

Statistical analyses were conducted in GraphPad Prism v10. Datasets were first tested for normal distribution using the Shapiro-Wilk test. Normally distributed datasets comparing only two groups were analyzed using Welch’s unpaired t-test. Differences among three groups were analyzed using one-way ANOVA, followed by Fisher’s protected LSD post hoc test comparing treatment groups to the control groups when the ANOVA was significant. The level of significance is * = *p* < 0.05, ** = *p* < 0.01 and *** = *p* < 0.001 and the results are presented as mean ± SD.

**Table 1:** List of antibodies used

Antibody	Host	Dilution	Vendor	Cat #	Technique
Vimentin (Reference)	Chicken	1:200	Sigma Aldrich	AB5733	ICC, IHC, WB
Vimentin RV202 clone	Mouse	1:200	Abcam	ab8978	IHC
Vimentin 84-1 clone	Mouse	1:200	Thermo Fisher	H00007431-M08J	IHC, PLA, ICC, WB
Aβ_42_	Rabbit	1:200	Thermo Fisher	700254	IHC
S100β	Rabbit	1:100	Novus Biologicals	NBP1-87102	ICC
GFAP	Chicken	1:400	Abcam	ab4674	ICC
GFAP	Rabbit	1:200	DAKO	Z0334	ICC
α-synuclein p-S129 (EP1536Y)	Rabbit	1:200	Abcam	ab209422	IHC, PLA
α-synuclein p-S129 (81 A)	Mouse	1:200	Abcam	ab184674	IHC
α-synuclein p-S129 (MJF-R13)	Rabbit	1:200	Abcam	ab168381	IHC
Anti-Tau (phospho T231)	Rabbit	1:200	Abcam	ab151559	IHC
Anti-Tau (phospho AT8)	Rabbit	1:200	Abcam	ab210703	IHC

## Results

### Intracellular deposits of 84-1 vimentin are present in the AD and PD brain

To gain insights into previously undetected pools of vimentin in the AD and PD brain, we utilized the vimentin antibody clone 84-1, originally developed for its preferential affinity for a distinct form of vimentin expressed on the surface of sarcoma cells over the intracellular filamentous vimentin [39]. First, we tested conventional vimentin antibodies to serve as a representative benchmark for comparison. For that, a chicken polyclonal antibody and a mouse monoclonal antibody were selected, each chosen for their high frequency of references in the literature. Fluorescent immunohistochemistry (IHC) of human brain sections revealed that both antibodies strongly label endothelial vimentin in the vessel wall, but that the polyclonal antibody was more comprehensive (Supplementary Fig. S1a). Because of that, and because the mouse antibody did not correspond well to the 84-1 signal (Supplementary Fig. S1b), the chicken polyclonal antibody (hereafter referred to as Ref. vimentin) was chosen as the reference antibody.

**Fig. 1:**
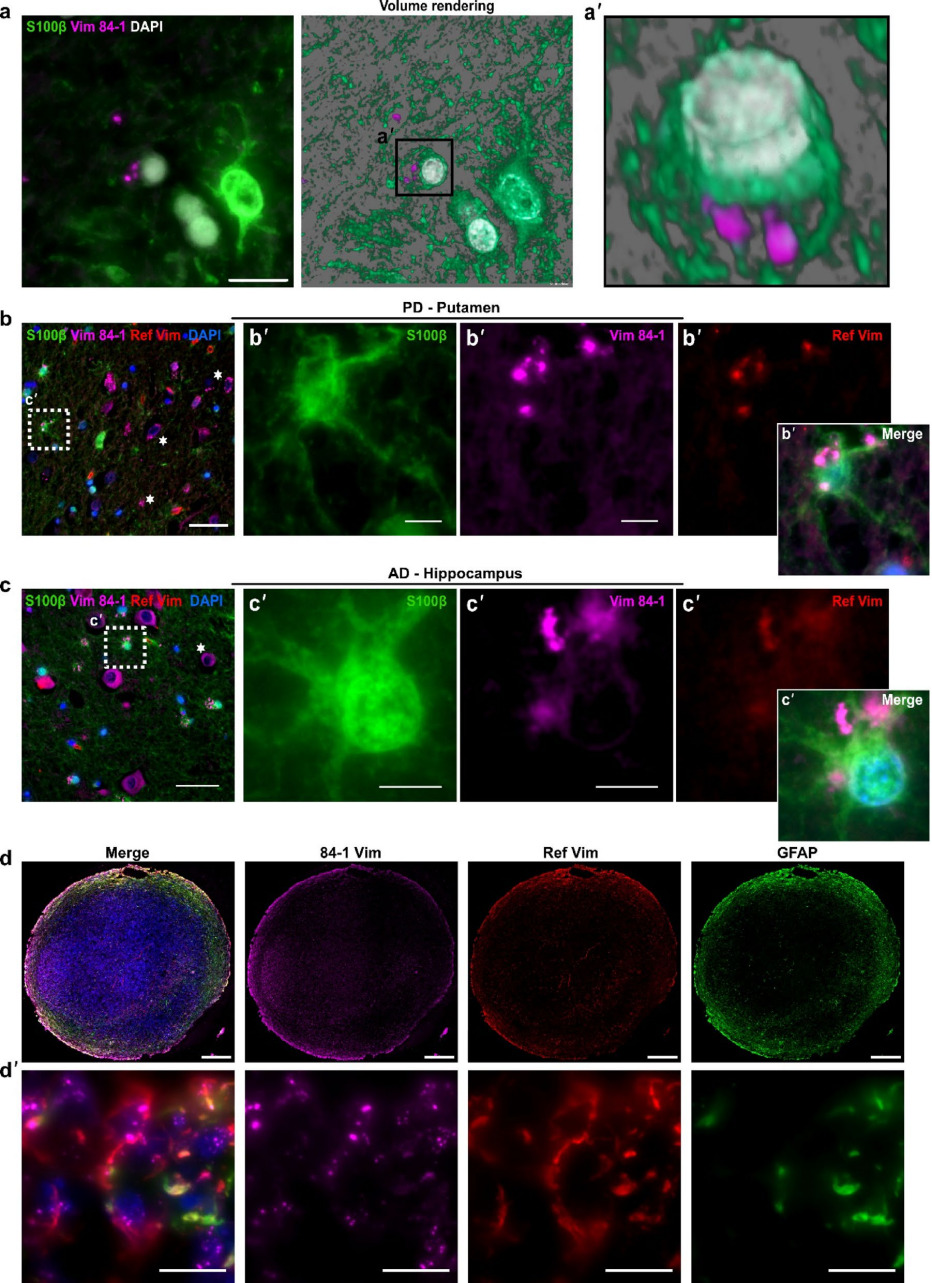
The 84-1 anti-vimentin antibody identifies a distinct form of vimentin in the human brain. **a** Representative 3D rendering of distinct intracellular vimentin deposits situated within a cell (**a’**) in 84-1 vimentin-stained brain sections. **b** and **c** Vimentin aggregates accumulate within cells (stars) and in S100β-positive astrocytes (box) in the striatum of PD patients (**b**) and the hippocampus of AD patients (**c**). **d**, Cortical organoids exposed to Aβ_42_ soluble aggregates show similar intracellular vimentin deposits that are detected by 84-1vimentin (Magenta), but not with GFAP (green) or Ref. vimentin antibodies (red). Scale bars: 10 μm in (**a**), (**b’**), (**c’**), and (**d’**), 40 μm in (**b**) and (**c**), and 200 μm in (**d**)

Next, we assessed the distribution and expression of 84-1vimentin in human striatum sections from PD patients and hippocampus sections from AD patients, along with age-matched controls. Indeed, the 84-1 staining revealed unique vimentin features in diseased conditions. Brains from AD and PD patients contained distinct, round vimentin deposits (Fig. 1a and a’) that appeared intracellularly across the investigated brain regions (Fig. 1b-c), often co-localizing with S100β + astrocytes (Fig. 1b’-c’). The Ref. vimentin antibody only faintly stained some of the 84-1^+^ deposits (Fig. 1b-c), suggesting a unique affinity of the 84-1 clone towards this vimentin form. Notably, the 84-1^+^ deposits were not detected in control sections, while 84-1 showed clear reactivity in S100 + astrocytes in both the striatum and hippocampus (Supplementary Fig. S2a, b). To further validate the presence of vimentin deposits, 37-week-old cerebral organoids treated with Aβ_42_ soluble aggregates were stained with the 84-1 and Ref. vimentin, in combination with the astrocytic marker GFAP. The staining showed a wide distribution of astrocytes in organoids, as shown by the GFAP and Ref. vimentin co-expression (Fig. 1d). More importantly, the 84-1 clone again revealed a different population of vimentin structures (Fig. 1d’), highly resembling the perinuclear accumulations in the brain tissue. Taken together, these findings indicate that the anti-vimentin [84-1] antibody can detect alternative vimentin forms in the diseased brain, highlighting limitations of conventionally used vimentin markers.

### 84-1 vimentin exhibits broad immunoreactivity across different brain regions

**Fig. 2:**
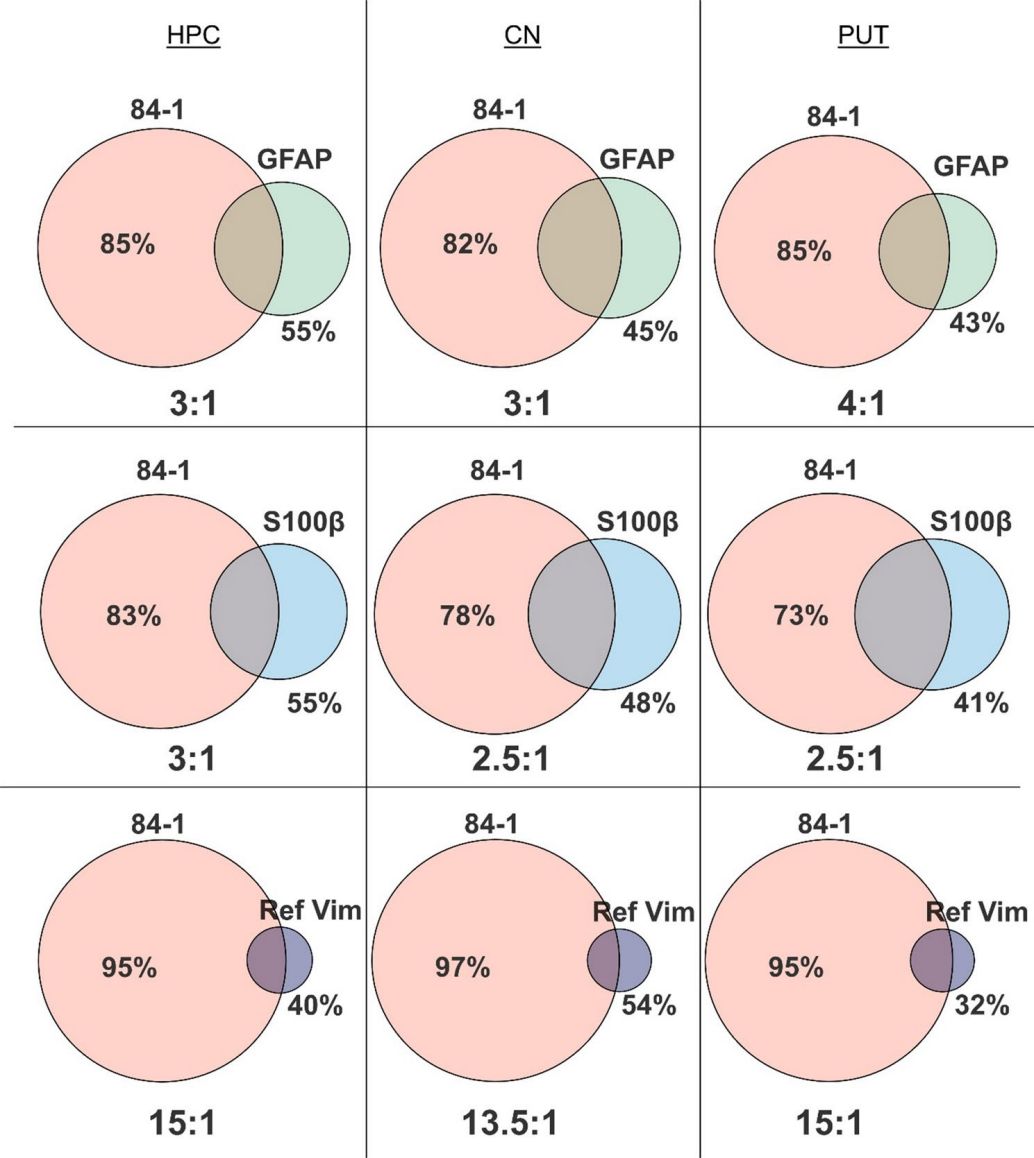
84-1 exhibits a broader labeling pattern and more structural coverage than conventional astrocytic markers. Analysis of human brain Hippocampus (HPC), Caudate Nucleus (CN) and Putamen (PUT), demonstrating the percentage of the non-overlapping structures and the labeling coverage ratio between each co-labeled marker pair: 84-1 (Pink) - GFAP (green), 84-1 (Pink) - S100β (Blue) and 84-1 (Pink) - Ref. vimentin (Violet). *n* = 5 individuals, Venn diagrams represent the ratio of the mean total area stained and the mean percentage overlap. The full list of analysis results can be found in Supplementary Table S3-5

When we examined the 84-1 vimentin^+^ structures, we frequently detected 84-1 signal in areas where GFAP and S100β reactivity was absent, despite vimentin being traditionally associated with astrocytes in the adult brain. This prompted us to characterize the brain distribution of the 84-1 vimentin form in relation to classical astrocytic markers. To achieve that, 84-1 immunoreactivity was examined in the caudate nucleus (CN) and putamen (PUT) of the striatum as well as the hippocampus (HPC) of healthy brain tissue, and the sections were co-stained with the astrocytic markers GFAP or S100β or the Ref. vimentin antibody. Clone 84-1 consistently showed a stronger and more inclusive immunoreactivity patterns across all the studied regions (Fig. 2, Supplementary Table S3-5). Colocalization analysis revealed that the vast majority of 84-1 vimentin is expressed outside the astrocytic structures that are defined by the three co-tested markers. For instance, traditionally marked astrocytes could be attributed to a maximum of 22% of the 84-1 positive area in the CN, while most of the staining was unique to 84-1 (82% unique − 18% overlap with GFAP, 78% unique − 22% overlap with S100β, and 97% unique − 3% overlap with Ref. Vimentin). In the HPC, 84-1 co-localized with approximately half of the astrocytic signal detected by the classical markers (45% overlap with GFAP, 45% overlap with S100β, and 60% overlap with Ref. vimentin). Additionally, the Ref. vimentin immunoreactivity showed the smallest overlap with the 84-1 signal. In the putamen, 84-1 covered an area approximately 15 times larger than that of Ref. vimentin (Fig. 2), demonstrating again that conventional antibodies recognize only a subset of the expressed protein.

**Fig. 3:**
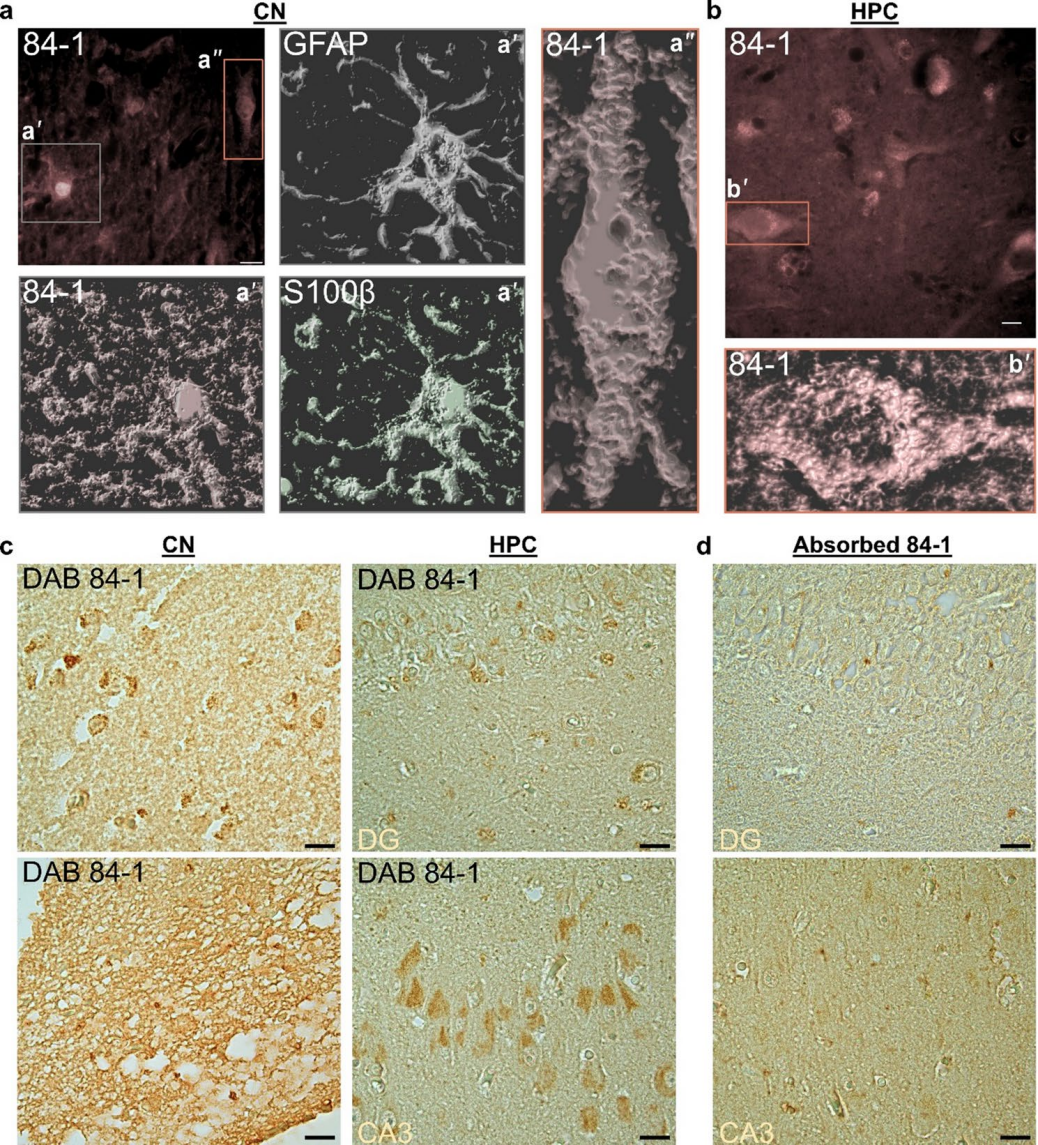
The 84-1 antibody labels distinct forms of vimentin in astrocytes and neurons. **a** The 84-1 antibody identifies astrocytic vimentin, but shows a distinct pattern compared to GFAP and S100β (**a’**). In addition, it identifies a form of vimentin in neurons (**a’’**) in the caudate nucleus. **b** Characteristic pyramidal neurons of the hippocampus stained with the 84-1 antibody display vacuole-like structures within the cytoplasm (**b’**). Multichannel source images of the rendering are shown in Supplementary Fig. S3. **c** Brightfield imaging of two examples from human Hippocampus (HPC) and Caudate Nucleus (CN), showing 84-1 vimentin DAB-positive signal. DG: dentate gyrus. **d** Control staining, following absorption of the 84-1 antibody with recombinant vimentin. Scale bars: 10 μm in (**a**) and (**b**), 20 μm in (**c**) and (**d**)

To further understand the characteristics of cells that express 84-1 vimentin, we performed volumetric imaging on a subset of representative regions. In GFAP/S100β double-positive astrocytes, 84-1 vimentin was predominantly located in the cell body around the nuclei, while the cellular projections were less defined. In contrast, the astrocytic ramifications were clearly outlined by both GFAP and S100β (Fig. 3a, a’, Supplementary Fig. S3a). A fraction of the 84-1 signal corresponded to brain cells that were negative for both GFAP and S100β, but had the characteristic shape of neurons with large nuclei (Fig. 3a’’). In these cells, 84-1 vimentin appeared in the form of vacuolar structures in the soma and was not detected in the neuronal axons (Fig. 3b, b’, Supplementary Fig. S3b). The neuronal identity was further confirmed upon examining the hippocampus, where 84-1 reactivity distinctively decorated the pyramidal and granule neurons of the dentate gyrus and the Cornu Ammonis (CA) (Supplementary Fig. S4). Fluorescence IHC clearly demonstrated the broad detection spectrum of brain vimentin using the 84-1 antibody. However, to verify the specificity of the fluorescent signal, we supplemented our analysis with the chromogenic DAB staining. Brightfield imaging of 84-1 DAB-stained sections confirmed the 84-1 reactivity pattern seen in the CN and the HPC, while ruling out potential cross-reactivity (Fig. 3c). The specificity of 84-1 was further supported by a clear abolishment of the DAB signal when the antibody was pre-absorbed with recombinant vimentin (Fig. 3d) or when the staining was performed in the absence of primary antibody (Supplementary Fig. S5a). Importantly, the DAB signal revealed the same differences in vimentin affinity between Ref. vimentin and 84-1 (Supplementary Fig. S5b). Thus, we can conclude that the observed 84-1 vimentin pattern is a true signal and that the clone has a selective affinity towards distinct vimentin forms.

### 84-1 brain vimentin is predominantly cleaved and shows a specific phosphorylation profile

**Fig. 4:**
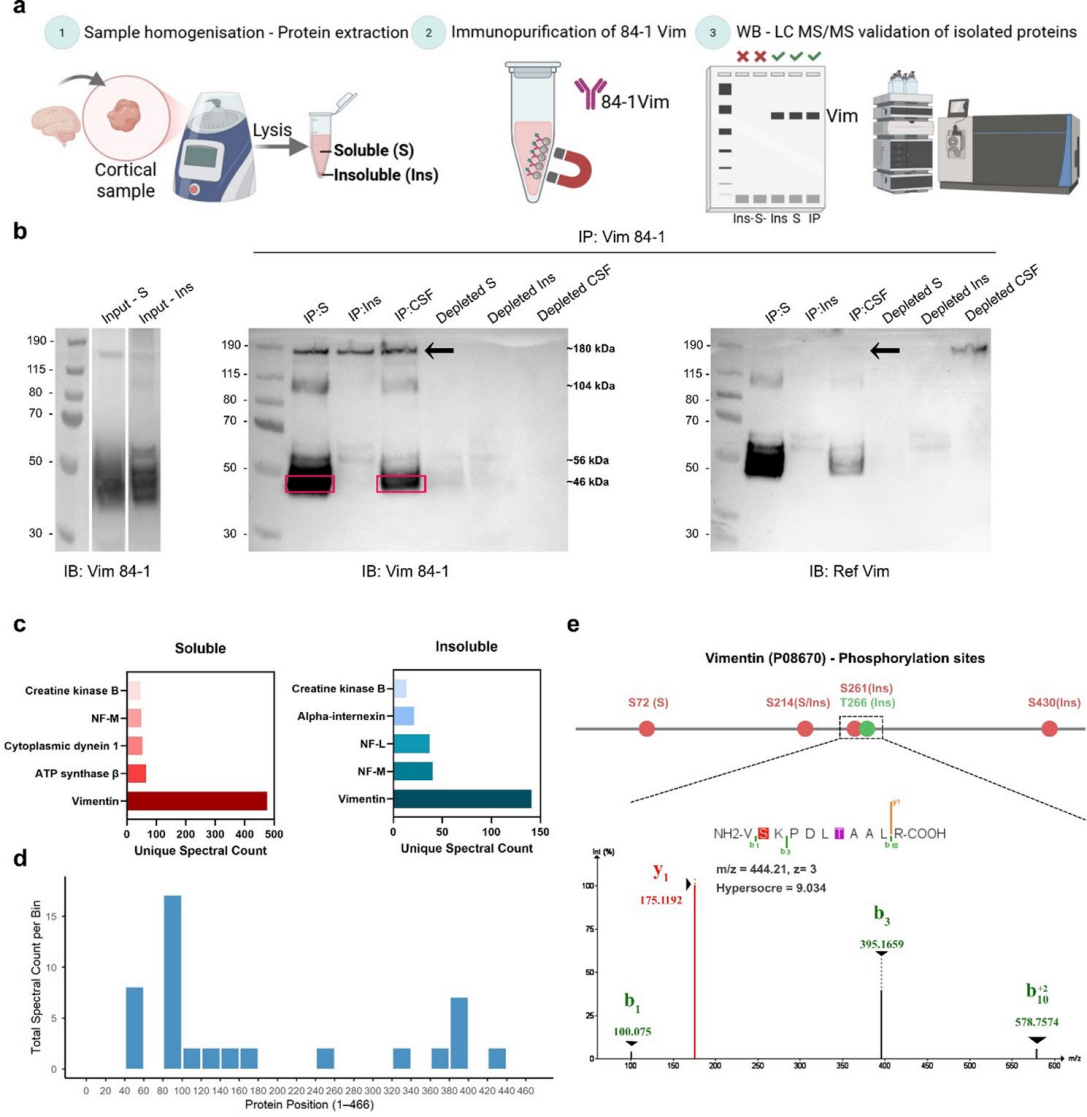
Immunoprecipitation analyses of brain homogenates demonstrate that the 84-1 antibody detects cleaved and specifically phosphorylated vimentin forms. **a** Schematic outline of 84-1 vimentin immunoprecipitation (IP) workflow prior to characterization with Western blot and LC-MS/MS analysis. **b** Immunoblots of 84-1 IP human brain homogenates (Lysis soluble fraction (S), Lysis insoluble fraction (Ins)) and human CSF. The input blots represent the expression levels of vimentin in the fractions used for IP. IP: Vim 84-1 blots show the experiment output probed with the same IP antibody (IB:84-1) and a Ref. vimentin antibody (IB: Ref. Vim), highlighting a smaller 46 kDa form of vimentin (Box) and clone-specific vimentin bands (Arrow), The full membranes and total protein loading controls are presented in supplementary Fig. S6. **c** List of the five top proteins detected in the proteomic analysis of the soluble and the insoluble IP isolated fraction, confirming isolation of vimentin. **d** Detection frequency (y-axis) of semi-tryptic peptide fragments plotted against their position in the vimentin sequence (x-axis). Each bin represents pooled counts from a 20 AA segment of vimentin. Only hits with ≥ 2 counts were included. **e** Detected Serine (S) and Threonine (T) vimentin phosphorylation in the soluble (S) and insoluble (Ins) fractions, box indicates the spectrum corresponding to the identified peptide with the modified residues highlighted

To investigate the molecular features of brain 84-1 vimentin in detail, we designed a workflow of target isolation by immunoprecipitation (IP) followed by proteomic analysis (Fig. 4a). Magnetic beads were coated with the 84-1 vimentin antibody and incubated with human brain homogenates in lysis buffer. This allowed for the selective isolation of soluble proteins from the supernatant fraction (S) and insoluble proteins from the pelleted fraction (Ins) that had high affinity for the 84-1 antibody. IP-isolated proteins were then analyzed by Western blotting or LC-MS/MS. Western blot analysis of the immunoprecipitated fraction indeed confirmed the identity of the isolated protein as vimentin (Fig. 4b). However, investigating the band sizes revealed that the majority of the brain 84-1 vimentin did not correspond to the canonical 56 kDa protein, but to a shorter 46 kDa vimentin proteoform (Fig. 4b, box). To validate this finding, we isolated 84-1 vimentin from human CSF using the same workflow. The predominant vimentin proteoform in CSF was also 46 kDa, confirming that it is an endogenous vimentin form and not an artifact of postmortem brain degradation. In addition to the 46 kDa and 56 kDa bands, several high molecular weight bands were detected, corresponding to reduction-resistant multimers. Notably, the largest form, a 180 kDa aggregate, appeared only when probing the membrane with the 84-1 clone, and not with the Ref. vimentin antibody (Fig. 4b, Arrow).

Next, we conducted a comprehensive proteomic analysis of the immunoprecipitated sample. Importantly, this analysis confirmed the pronounced enrichment of vimentin in the IP-isolated fraction (Fig. 4c, Data S1), verifying the specificity of the 84-1 antibody towards vimentin. To assess whether the isolated 84-1 vimentin underwent proteolytic processing in vivo, the MS analysis was designed to include peptide fragments that would not conform to expected trypsin cleavage patterns. These semi-tryptic matches could represent pre-existing cleavages that took place before sample processing. Analysis showed that the Semi-tryptic fragments were predominantly clustered near the N-terminus of the protein around residues 80–100 on the full-length protein sequence (Fig. 4d). This clustering of semi-tryptic peptides is consistent with a protein that has been N-terminally cleaved into a proteoform of approximately 370 amino acids (AA), which corresponds well to the 46 kDa vimentin band detected on the immunoblots. Additionally, post-translational modification profiling identified several previously reported phosphorylated sites, including Ser-72, Ser-214, and Ser-430. However, it also revealed two undescribed modifications at Ser-261 and Thr-266 (Fig. 4e, Supplementary Table S6). These phosphorylated subunits appeared only in the insoluble pellet fraction, suggesting a possible association with the intracellular vimentin deposits detected by IHC.

### 84-1 vimentin co-localizes with αSyn and Aβ deposits in the human brain

**Fig. 5:**
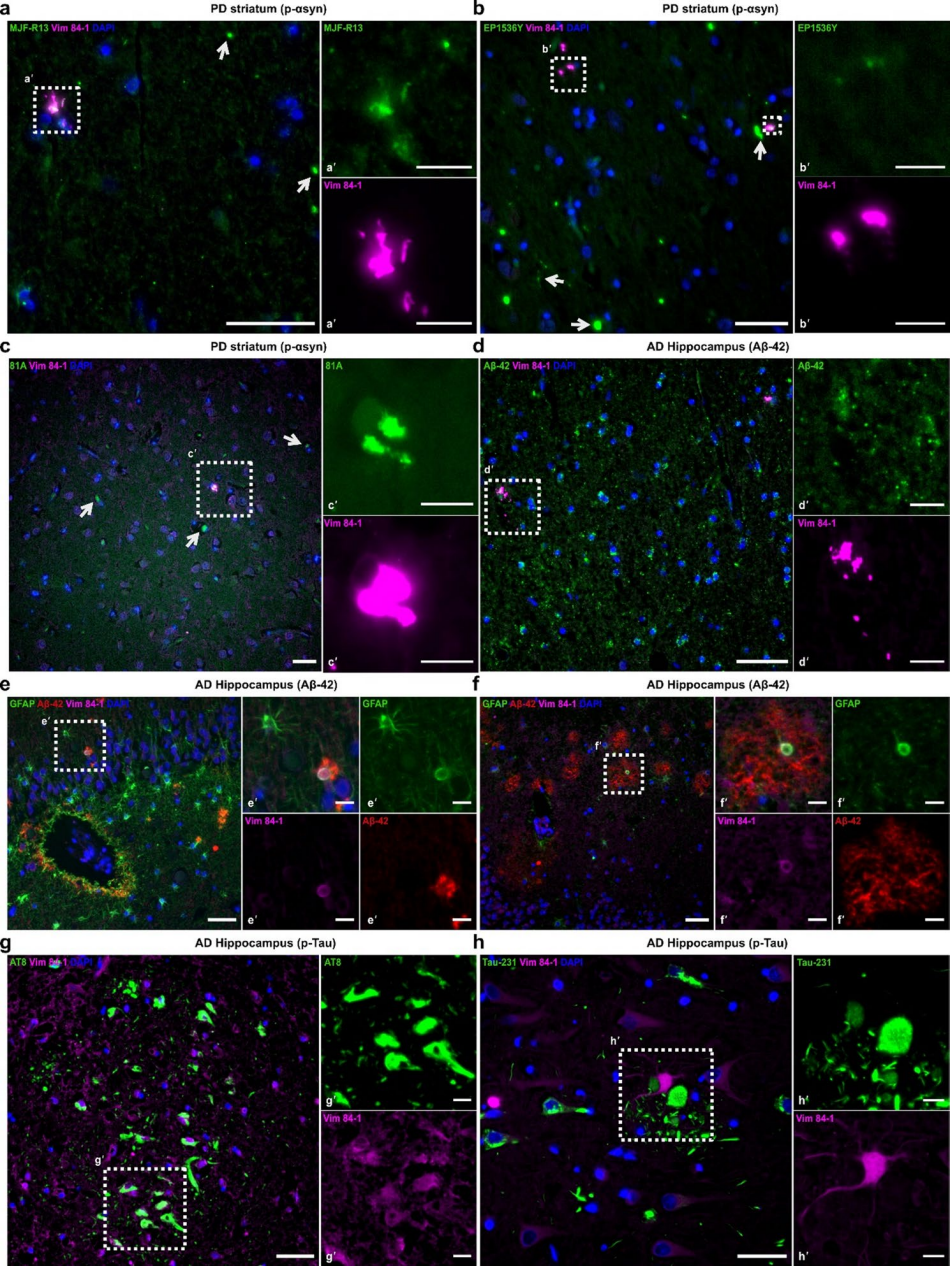
84-1-vimentin is associated with pathological protein aggregates in the AD and PD brain. **a** IHC of human PD striatum, using the 84-1 vimentin antibody in combination with the MJF-R13 anti p-αsyn^S129^ antibody. Arrows indicate characteristic Lewy bodies, and the box highlights 84-1 vimentin overlap. **b** Co-labelling of 84-1 and the EP1536Y anti p-αsyn^S129^ antibody. Arrows indicate Lewy bodies/neurites, and boxes represent double-positive aggregates. **c** Co-labelling of 84-1 and the 81A anti p-αsyn^S129^ antibody. Arrows indicate characteristic Lewy bodies, and the box represents double-positive aggregates. **d** IHC of human AD hippocampus displays Aβ_42_ deposits around 84-1-vimentin. **e**-**f** 84-1-positive staining around dense (**e**) and diffuse Aβ_42_ plaques (**f**). Box shows spherical 84-1-vimentin structures in the center of an Aβ_42_ plaque, co-localizing with the astrocytic marker GFAP. **g**-**h** diffuse 84-1 vimentin expression in neurons carrying AT8^+^ tangles (**g’**) and strong astrocytic expression of 84-1-vimentin in regions of high p-Tau231 (**h’**). Scale bars, 40 μm in (**a**-**h**), 10 μm in (**a’**-**h’**)

We next sought to investigate whether there is an association between 84-1 vimentin and pathological protein deposits in AD and PD. Sections from PD striatum were co-stained with 84-1 and p-αsyn antibodies, while sections from AD hippocampus were stained with 84-1 in combination with antibodies against either Aβ_42_ or p-Tau. In the PD brain, three widely used p-αsyn^S129^ antibodies made in rabbit (EP1536Y, MJF-R13) or mouse (81A) were able to successfully label Lewy bodies and Lewy neurites (Fig. 5a-c). Interestingly, a fraction of the 84-1 vimentin signal encapsulated the MJF-R18 positive aggregates (Fig. 5a). The same pattern was detected using the two other p-αsyn^S129^ antibodies; EP1536Y (Fig. 5b) and 81 A (Fig. 5c), both showing a p-αsyn positive core with a thick coat of 84-1 vimentin.

In the AD brain, 84-1 vimentin appeared around smaller accumulations of Aβ_42_ (Fig. 5d) and formed spherical structures at the core of both dense (Fig. 5e) and diffuse Aβ plaques (Fig. 5f), which were also positive for GFAP. AT8^+^ neurofibrillary tangles, spanning the AD hippocampus, did not colocalize with the 84-1 vimentin deposits and the signal appeared diffuse within the affected neurons (Fig. 5g). However, astrocytes with very high 84-1 vimentin expression were frequently found in regions of concentrated p-Tau231 reactivity (Fig. 5h), a tau epitope that is known to be phosphorylated early during AD development [1]. Taken together, our data support a relationship between the unique 84-1 vimentin form and pathological protein aggregation in the human brain.

### A significant increase in free, extracellular 84-1 vimentin is associated with AD/PD pathology

**Fig. 6:**
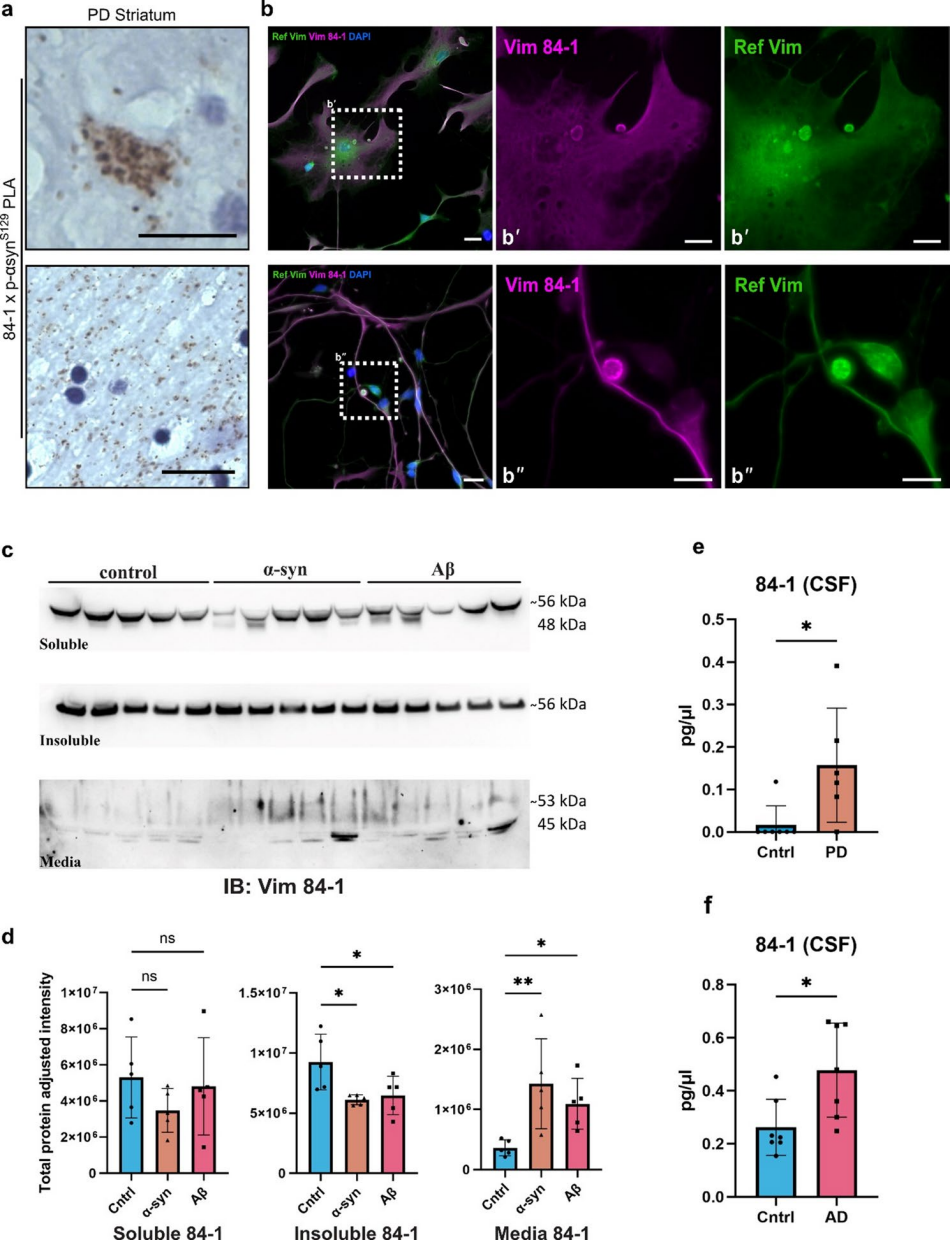
Released 84-1-vimentin represents a potential biomarker for AD and PD pathology. **a** PLA with the 84-1 vimentin antibody and the EP1536Y p-αsyn^S129^ antibody revealed positive intracellular and scattered puncta, confirming direct interactions between vimentin and p-αsyn^S129^. **b** Representative ICC images of vimentin 84-1 positive astrocytes (**b’**) and zombosomes (**b’’**) in culture, co-stained with the Ref. vimentin antibody. **c** Immunoblots showing the expression levels of 84-1 vimentin in the soluble and insoluble fraction of cell lysates, as well as in conditioned media from Aβ/αsyn-exposed astrocyte cultures and control cultures. **d** Western blot quantification of vimentin expression levels, normalized to total proteins for each loaded sample (*n* = 5, one-way ANOVA followed by protected Fisher’s LSD post-hoc test. Data shown as mean ± SD. ns: non-significant, *p < 0.05, **p < 0.01). **e** and **f** ELISA analysis of vimentin levels in human CSF samples from PD and age-matched controls (**e**), as well as AD and age-matched controls (**f**), (*n* = 7, Two-tailed unpaired t-test. Data shown as mean ± SD. *p < 0.05). The full membranes and total protein loading controls are shown in Supplementary Fig. S7. Scale bars: 20 μm in (**a**), (**b**), 10 μm in (**b’**), (**b’’**)

Given the pronounced co-localization of vimentin 84-1 and pathological protein deposits, we were prompted to investigate this disease association in more detail. First, proximity ligation assay (PLA) was used to validate close interaction between vimentin and pathological αsyn in the PD brain. Indeed, 84-1 vimentin and EP1536Y p-αsyn^S129^ PLA analysis of PD striatum, revealed diffuse clusters of intracellular puncta (Fig. 6a), corresponding to those observed with fluorescent IHC. Having confirmed interaction between vimentin 84-1 and amyloid aggregates in vivo, we next sought to elucidate the basic features and outcomes of this interaction using a simplified in vitro system. To achieve that, we performed immunocytochemistry (ICC) fluorescent staining on monocultures of human induced pluripotent stem cell (hiPSC)-derived astrocytes using either the 84-1 clone or the Ref. vimentin antibody. Comparison between the two staining patterns revealed the same discrepancies observed in the brain sections, confirming the limitations in capturing all vimentin proteoforms using a single antibody. Both vimentin antibodies successfully stained the overall structure of the astrocytes and displayed notable overlap, suggesting a shared target protein identity (Fig. 6b), but a clear preference for different forms of the protein. Ref. vimentin ICC appeared diffuse, primarily localized to the cytoplasmic pool, but also delineated segments of the skeletal vimentin. In contrast, 84-1 immunoreactivity was broader, showing sharper and more defined fibrillar structures that efficiently highlighted the astrocytic vimentin skeleton (Fig. 6b’). Moreover, the 84-1 antibody clearly outlined zombosomes, which were recently reported to be migrating vehicles shed from astrocytes [10] (Fig. 6b’’).

To simulate AD and PD pathology in the culture dish, astrocytes were exposed to either sonicated αSyn or Aβ fibrils for three days, and then cultured in fibril-free media for an additional period of four days (3 + 4d) [22, 35, 37]. Cells were then lysed and fractionated by centrifugation into soluble and insoluble protein pools. Additionally, the conditioned media from treated and control cultures were collected. Western blot analysis using the 84-1 antibody detected the same cleaved ~ 46 kDa vimentin (in addition to the canonical vimentin form) both in the soluble lysate fraction and in cell media, indicating that the culture system can mirror in vivo brain vimentin processing (Fig. 6c). The soluble pool of vimentin showed no significant difference across treatment groups (Fig. 6d). However, both Aβ and αSyn treated cultures displayed significantly lower levels of the more compact filamentous vimentin found in the insoluble protein pool (Fig. 6d). This decrease was accompanied by a substantial increase in vimentin released into the media of pathology-bearing astrocytes (Fig. 6d), suggesting active secretion or redistribution of the cells’ vimentin reservoir.

CSF levels of disease-associated proteins are believed to reflect the extracellular disease environment and are commonly investigated to establish efficient diagnostic markers for neurodegenerative diseases. Thus, we measured 84-1 vimentin levels in CSF samples from AD and PD patients, as well as from age-matched healthy controls via ELISA. The CSF analysis showed that our in vitro results recapitulated clinically relevant, disease-associated processes, as CSF levels of vimentin 84-1 were significantly elevated in both PD (Fig. 6e) and AD (Fig. 6f). Collectively, our results introduce the anti-vimentin clone [84-1] antibody as a new tool to study the diversity of brain vimentin. We also demonstrate a link between 84-1vimentin and pathological changes in AD and PD, suggesting that this form of vimentin can serve as a potential disease biomarker.

## Discussion

Dysregulation of the vimentin network is tightly linked to cellular damage and neurodegeneration [27]. Despite that, knowledge of how vimentin is modified in the AD and PD brain is very limited. This is largely due to the lack of selective tools to study vimentin modifications with precision.

Here, we provide a method for identifying a broader population of vimentin in the human brain, using the isoform-specific antibody clone 84‑1. Our results indicate that the identified vimentin population consists predominantly of a non-canonical, cleaved form of vimentin that is released into the extracellular space in the presence of pathological protein aggregates. The 84‑1 clone is a mouse monoclonal antibody raised against full-length vimentin to specifically recognize a pathological form of cell-surface vimentin (CSV) on cancer cells undergoing epithelial–mesenchymal transition, which reflects their metastatic potential [39]. Previous studies using this antibody clone reported high affinity and relative specificity towards CSV, without cross-reactivity with non-cancerous epithelial or mesenchymal cells [25, 40], making it an ideal biomarker for tumor detection assays [51]. We here present the first characterization of this vimentin form in the human brain. Our results demonstrate that the 84-1 antibody does not retain the same CSV selectivity in the brain as for cancer cells in the periphery, since it reacted significantly with intracellular vimentin. However, this broad immunoreactivity profile can be attributed to its unique ability to recognize modified forms of the protein. Extracellular vimentin is produced after intracellular vimentin filaments disassemble into smaller soluble oligomers, in a process tightly regulated by PTMs [32]. The modified extracellular vimentin can then appear as cell‑surface vimentin, free/secreted vimentin, or vesicle‑bound vimentin that may be taken up by neighboring cells [7, 53]. This diversity reflects a highly heterogeneous pool of vimentin, poorly captured by the antibodies currently used to study neurodegenerative changes. This limitation may explain why vimentin has not developed into a reliable AD or PD biomarker, despite its clear involvement in disease progression. The reference vimentin antibody used here has previously been reported to show no correlation between vimentin expression and neuropathological features of PD [49]. However, proteomic analysis has identified a link between CSV vimentin and early tau hyperphosphorylation in mice [34]. In line with these findings, we demonstrate here that 84-1 vimentin, but not Ref. vimentin, correlates with pathological features in the AD and PD brain. Moreover, we show that the 84-1 antibody detects a significant increase in vimentin release into CSF in both AD and PD patients, compared to age-matched controls. These observations were recapitulated in vitro, where cell media from human astrocytes harboring pathological protein aggregates had significantly higher levels of 84-1 vimentin than the media from control cultures.

Cleaved forms of vimentin have been associated with various pathological conditions. For example, it has been shown that vimentin undergoes proteolytic processing during apoptosis, as Caspase-3 cleavage removes the N-terminal end of the protein, resulting in a 46 kDa fragment [6]. Here, we show that a high proportion of the brain vimentin recognized by 84-1 consists of 46 kDa fragments. Additionally, the semi-tryptic peptide fragments were clustered around AA 80 and 100, where caspase-3 is known to cleave vimentin at AA85 [6]. Hence, 84-1 vimentin in the diseased brain may stem from apoptotic cells. Accordingly, the cleaved form of vimentin has been identified in inflammatory diseases, such as in synovial tissue damage [46]. However, it was also reported in healthy chondrocytes, suggesting non-pathological processing mechanisms [23].

Calpains are other enzymes involved in vimentin cleavage. Calpains cleave the N-terminal head domain of vimentin [47], a region essential for IF assembly [48]. Thus, headless vimentin cannot polymerize into filaments, limiting it instead to monomers, dimers, or tetramers [15]. These cleaved forms can also interfere with the polymerization of full-length vimentin and destabilize existing IF networks [33]. The soluble 46 kDa vimentin species detected by the 84-1 antibody can be cleavage products that polymerized, but failed to form organized IF networks. This may explain the stable multimeric bands detected in the IP-isolated fraction. It is possible that these multimeric forms can function as a nucleus for aggregation, leading to secondary misfolding of physiological vimentin and intracellular deposition.

Many lines of evidence identify neuroinflammation as a driving force of neurodegenerative disease pathology. Being a key modulator of the astrocytic inflammatory response, vimentin can significantly affect disease progression [9]. For example, regulatory T-cell suppression of astrocyte activation has been shown to improve recovery after spinal cord injury, by downregulating vimentin. The release of IL-10 by T-cells activates the astrocytic STAT3-Protein Kinase C-vimentin signaling axis to dampen astrogliosis [26]. Moreover, extracellular vimentin can activate NF-κB signaling in macrophages, leading to the release of pro-inflammatory TNF-α and IL-6 [20]. Importantly, the released vimentin can also bind to Toll-like receptor 4 (TLR4) and thereby, function as a damage-associated molecular pattern (DAMP) [45]. TLR4 activation is tightly associated with neurotoxic inflammation and aggravates microglial responses in neurodegenerative disease [2], indicating a clear correlation between vimentin and inflammation-driven pathology.

Another major feature of AD and PD is the characteristic deposition of Aβ, tau, and αSyn that gradually spreads across the brain, causing escalating symptoms. However, the mechanistic relationship between pathological changes and other disease-associated proteins, such as vimentin, is poorly understood. Here, we show that the levels of 84-1 vimentin in CSF is significantly increased in AD and PD individuals, as well as in Aβ/αSyn-treated astrocyte medium. 84-1 vimentin was also detected as aggregating intracellular clusters that co-localize with Aβ and αSyn deposits. This suggests negative synergies between pathological protein aggregates and IF aggregates in neurodegeneration. It has been shown previously that filamentous cytoskeletal proteins can misfold into large deposits that physically impair mitochondrial trafficking and can result in metabolic and oxidative stress. This has been reported in diseases like giant axonal neuropathy (GAN), where the perinuclear accumulations of intermediate filaments (IFs), including vimentin, occlude the cellular space [3, 17]. In Alexander disease, mutations in GFAP IFs can also lead to the formation of intracellular Rosenthal fibers, resulting in astrocytic dysfunction and eventually cognitive and motor impairments [44].

In conclusion, our data reveal new roles for modified vimentin in neurodegenerative disease pathology and identify 84-1 vimentin as a promising biomarker and a possible treatment target in AD and PD.

## Supplementary Information

Below is the link to the electronic supplementary material.

Supplementary Material 1

Supplementary Material 2

## Data Availability

All generated data are available from the corresponding author on reasonable request.

## Abbreviations

AD: Alzheimer’s disease
PD: Parkinson’s disease
CSF: Cerebrospinal fluid
αSyn: alpha-synuclein
Aβ: Amyloid-beta
Ref. Vimentin: Reference Vimentin
PTM: Post-translational modification
ICC: Immunocytochemistry
IHC: Immunohistochemistry
IP: Immunoprecipitation
S: Soluble protein fraction
Ins: Insoluble protein fraction
AA: Amino acid
PLA: Proximity ligation assay
hiPSCs: Human induced pluripotent stem cells
IF: Intermediate filament
TLR4: Toll-like receptor 4
DAMP: Damage-associated molecular pattern
