# Supplementary material for: Characterization of a distinct form of vimentin in the neurodegenerative brain
Source: Acta Neuropathol Commun. 2026 May 22;14:111. doi: 10.1186/s40478-026-02324-9 (PMC13196128; doi:10.1186/s40478-026-02324-9)
Supplement: Supplementary file 2 — Supplementary Material 2 [file 40478_2026_2324_MOESM2_ESM.pdf]

Acta Neuropathologica Communications

Supplementary Information (SI) for

Characterization of a distinct form of vimentin in the neurodegenerative brain

Abdulkhalek Dakhel[1], Johanna Vestin[1], Vilmantas Giedraitis[1], Dag Nyholm[2], Martin Ingelsson[1, 3, 4], Anna Erlandsson[1]*

[1]Uppsala University, Department of Public Health and Caring Sciences; Molecular Geriatrics, Uppsala, Sweden

[2]Department of Medical Sciences, Neurology, Uppsala University, Uppsala, Sweden.

[3]University Health Network, Krembil Brain Institute, Toronto, ON, Canada.

[4]Tanz Centre for Research in Neurodegenerative Diseases, Departments of Medicine and Laboratory Medicine & Pathobiology, University of Toronto, Toronto, ON, Canada.

*Correspondence: Anna Erlandsson (anna.erlandsson@uu.se), Uppsala University

Supplementary Table S1: Brain tissue donor information

	Age (± SD), Years	PMD* (± SD), Hours	Tau staging (No.)	αSyn deposits (No.)	
				Pos.	Neg.
Cntrl (n=8)	74.75 (± 2.82)	81.00 (± 36.14)	Pre-tangle a (8)	-	8
AD (n=4)	74.00 (± 3.46)	90.00 (± 36.00)	Braak Ⅴ (2)/ Braak Ⅵ (2)	-	4
PD (n=4)	75.75 (± 4.03)	84.00 (± 41.57)	Braak Ⅰ (2)/ Braak Ⅱ (2)	4	-

*PMD: Post-Mortem Delay

Supplementary Table S2: CSF donor information

Diagnosis	Cognitively Healthy (n=7)	AD (n=7)	Healthy (n=7)	PD (n=7)
Age (± SD), Years	80.80 (± 2.68)	80.60 (± 5.03)	66.86 (± 7.52)	68.00 (± 10.13)
MMSE* (± SD)	29.20 (± 0.84)	23.20 (± 2.17)	-	-
Sampling time after diagnosis (± SD), years	-	0	-	10.00 (± 5.60)

*MMSE: Mini-Mental State Examination (0-30)

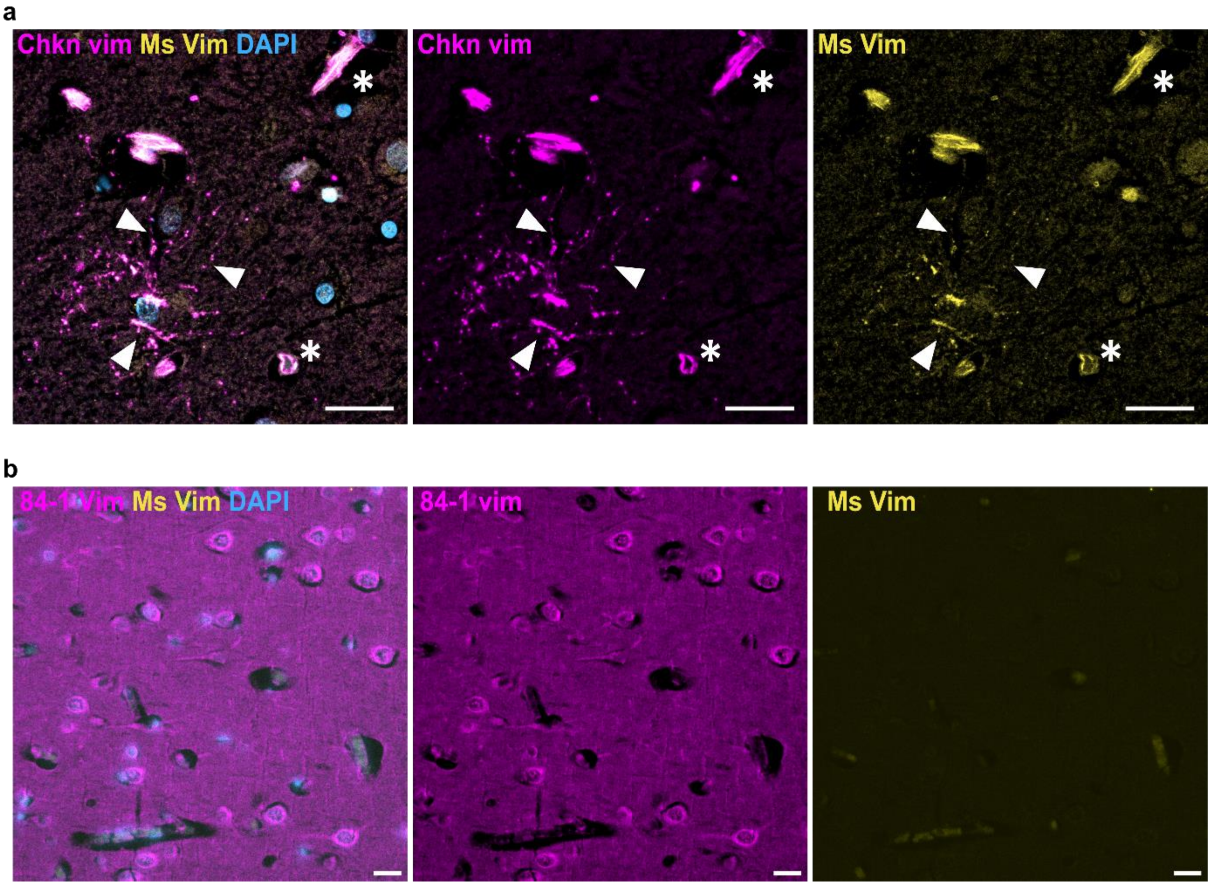

Supplementary Figure S1: a. Comparison of the IHC staining pattern of a polyclonal chicken and a monoclonal mouse anti-vimentin antibody. Stars represent endothelial vimentin, and arrows highlight astrocytic projections. **b.** Representative images showing the stronger and more comprehensive signal of the 84-1 vimentin antibody compared to the monoclonal mouse vimentin antibody. Scale bars: 20 µm.

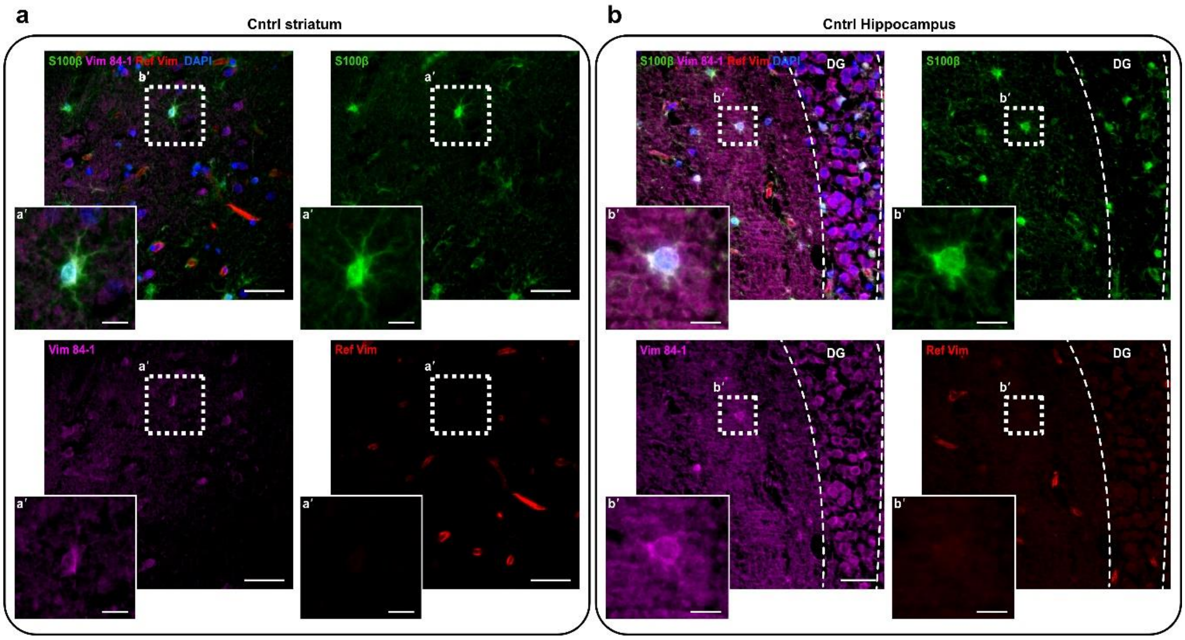

Supplementary Figure S2: Representative examples of human striatum (**a**) and hippocampus (**b**) of control brain sections stained with the anti-vimentin clone 84-1 antibody in combination with a reference anti-vimentin antibody and the astrocytic marker S100β. Boxes (**a’, b’**) highlight individual astrocytes. Scale bars: 40 µm in a-b, 10 µm in a’-b’.

Supplementary Table S3: Characterization of the overlapping patterns of S100b - 84-1

	Patient No.	Total area (mm2) S100β	Total area (mm2) 84-1	Percentage overlap S100β ∩ 84-1	Percentage overlap 84-1 ∩ S100β
HPC	Pt001	5.54	22.50	37.94	9.97
	Pt002	6.30	12.84	39.87	19.80
	Pt003	4.31	17.93	63.40	15.44
	Pt004	7.49	16.02	52.66	25.27
	Pt005	6.01	19.44	46.81	16.16
	**Mean (±SD)**	5.93 (± 1.16)	17.75 (± 3.63)	48.14 (± 10.34)	17.33 (± 5.66)
CN	Pt001	9.41	20.59	45.23	20.72
	Pt002	9.63	28.90	45.65	16.29
	Pt003	4.97	16.17	60.17	18.64
	Pt004	6.81	11.25	54.69	33.08
	Pt005	5.41	14.96	54.96	19.89
	**Mean (±SD)**	7.25 (± 2.19)	18.38 (± 6.76)	52.14 (± 6.50)	21.72 (± 6.56)
PUT	Pt001	16.44	31.63	43.72	23.33
	Pt002	11.20	50.54	51.37	12.67
	Pt003	12.87	37.24	51.42	19.47
	Pt004	19.98	36.81	73.05	39.88
	Pt005	17.63	40.65	75.02	37.25
	**Mean (±SD)**	15.62 (± 3.57)	39.37 (± 7.02)	58.92 (± 14.17)	26.52 (± 11.68)

Supplementary Table S4: Characterization of the overlapping patterns of GFAP - 84-1

	Patient No.	Total area (mm2) GFAP	Total area (mm2) 84-1	Percentage overlap GFAP ∩ 84-1	Percentage overlap 84-1 ∩ GFAP
HPC	Pt003	7.95	23.16	53.88	19.02
	Pt004	10.14	18.62	18.77	23.58
	Pt006	4.51	23.62	54.12	10.81
	Pt002	4.92	16.88	45.90	13.99
	Pt007	2.79	14.84	50.28	9.84
	**Mean (±SD)**	6.06 (± 2.94)	19.42 (± 3.86)	44.59 (± 14.81)	15.45 (± 5.79)
CN	Pt003	6.40	17.32	52.65	19.62
	Pt004	10.88	12.62	42.85	36.95
	Pt006	1.98	13.38	48.87	7.37
	Pt002	2.62	16.90	75.08	12.66
	Pt007	3.01	11.91	59.21	15.09
	**Mean (±SD)**	4.98 (± 3.72)	14.43 (± 2.51)	55.73 (± 12.34)	18.34 (± 11.30)
PUT	Pt003	18.06	42.11	53.65	26.86
	Pt004	17.47	41.52	64.35	27.26
	Pt006	4.28	42.99	54.32	5.97
	Pt002	4.72	48.48	70.20	7.84
	Pt007	4.17	22.97	41.62	7.83
	**Mean (±SD)**	9.74 (± 7.33)	39.61 (± 9.71)	56.83 (± 10.99)	15.15 (± 10.90)

Supplementary Table S5: Characterization of the overlapping patterns of Reference vimentin - 84-1

		Total area (mm2)		Percentage overlap	
	**Patient No.**	**Ref Vim**	**84-1**	**Ref Vim ∩ 84-1**	**84-1 ∩ Ref Vim**
**HPC**	Pt001	2.59	22.49	50.94	6.42
	Pt002	1.16	12.62	54.63	5.02
	Pt008	1.00	11.77	65.17	5.60
	Pt006	0.59	19.87	62.75	1.96
	Pt005	0.96	18.39	65.52	3.50
	**Mean (±SD)**	1.26 (± 0.77)	17.03 (± 4.66)	59.80 (± 6.62)	4.50 (± 1.78)
**CN**	Pt001	2.41	20.57	50.99	5.97
	Pt002	1.20	29.02	51.32	2.17
	Pt008	0.76	6.72	34.68	3.94
	Pt006	0.57	13.38	47.29	2.01
	Pt005	0.86	18.36	47.67	2.25
	**Mean (±SD)**	1.16 (± 0.73)	17.61 (± 8.31)	46.39 (± 6.80)	3.27 (± 1.70)
**PUT**	Pt001	3.75	31.58	56.60	6.77
	Pt002	2.18	49.71	59.32	2.93
	Pt008	1.55	23.12	50.87	3.54
	Pt006	2.99	35.33	98.55	10.79
	Pt005	1.18	36.55	73.91	2.63
	**Mean (±SD)**	2.33 (± 1.05)	35.26 (± 9.64)	67.85 (± 19.15)	5.33 (± 3.47)

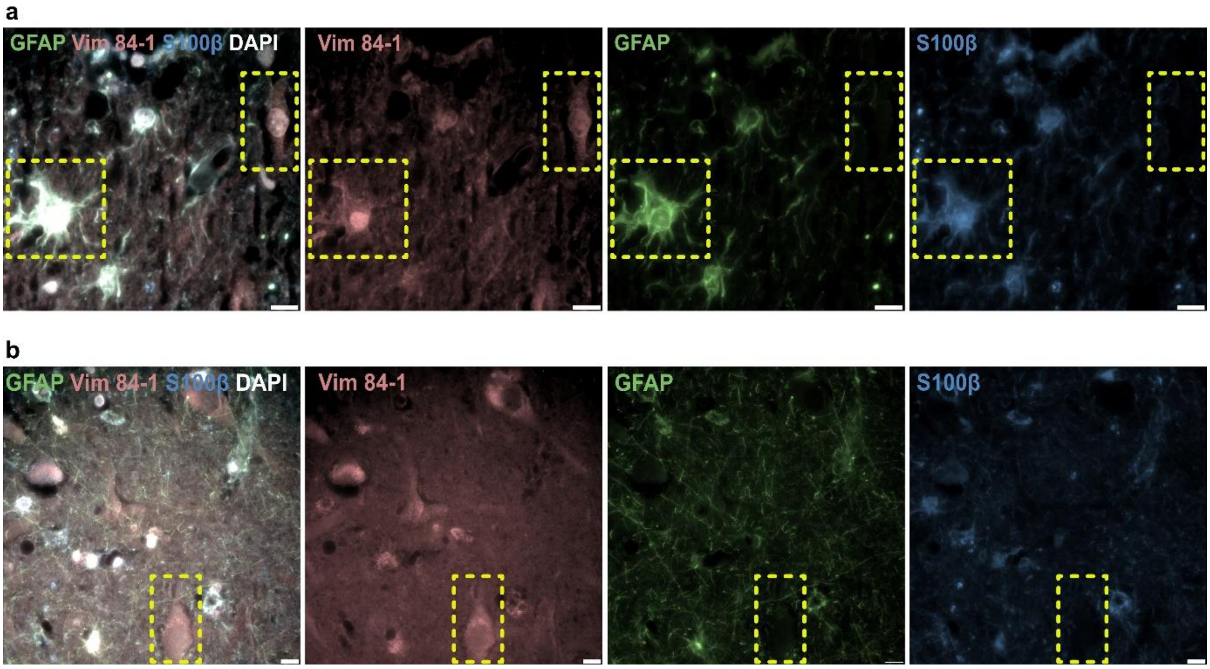

Supplementary Figure S3: Source Max-projection images of the volumetric 3D rendered images in Figure 3a (a) and Figure 3c (b). Boxes indicate the rendered cells shown in the figure. Scale bars: 10 µm.

a

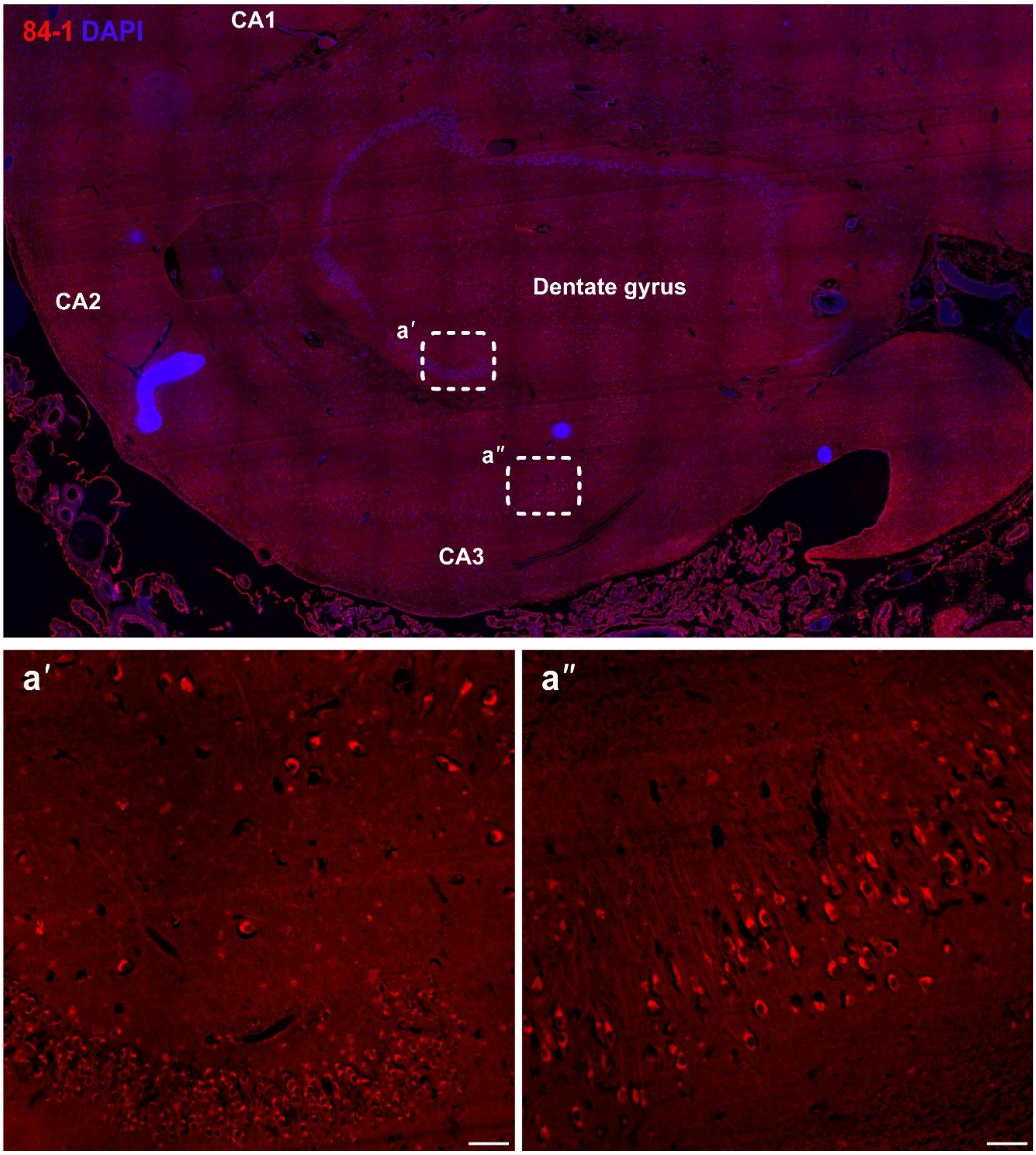

Supplementary Figure S4: A representative example of a scanned hippocampus region in the AD brain, showing a distinct immunoreactive pattern of 84-1 (red), selectively labeling vimentin accumulations in the cell bodies of pyramidal neurons in the CA region. Scale bars: 50 µm.

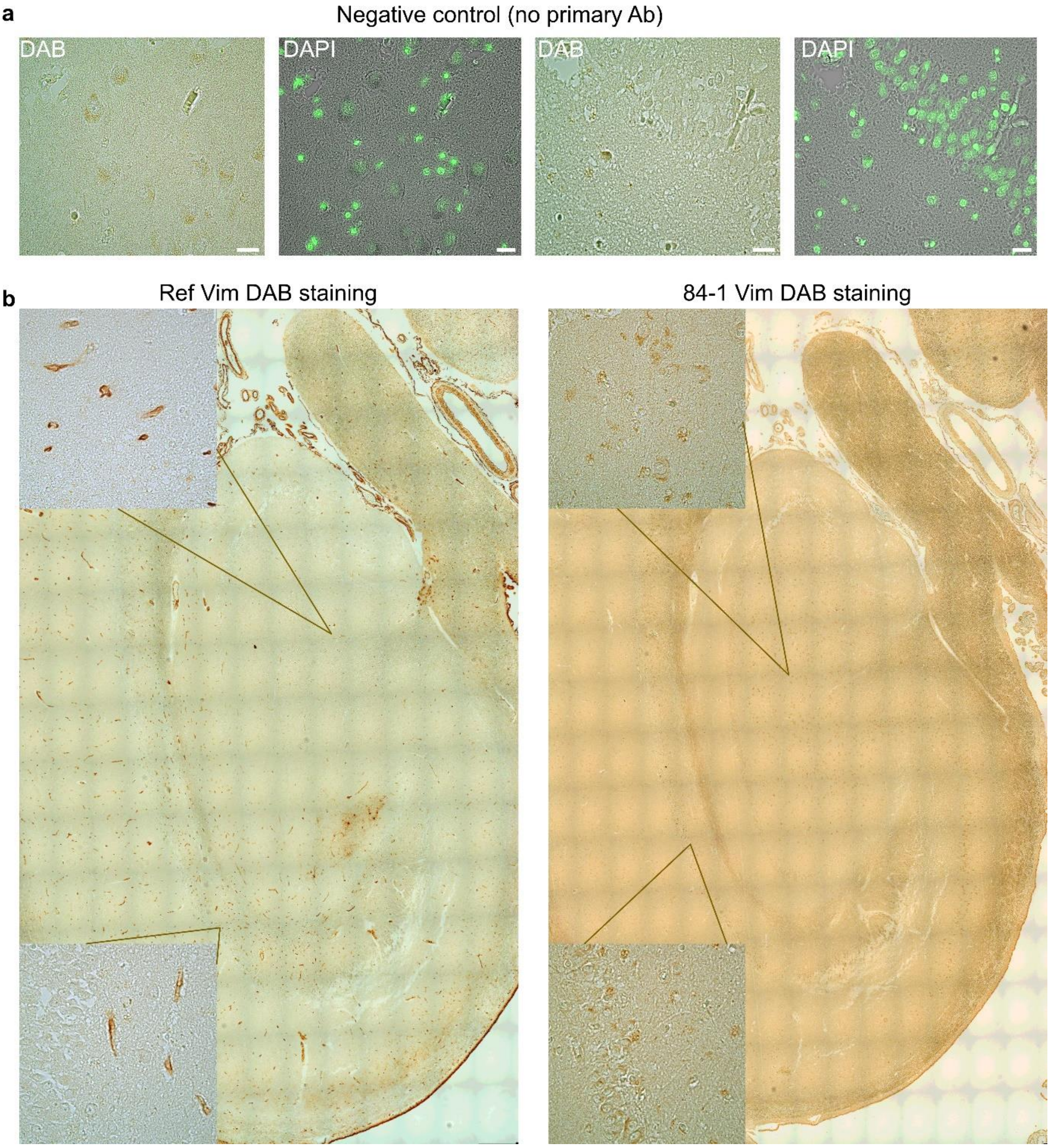

Supplementary Figure S5: a. DAB staining and corresponding DAPI nuclear staining, showing the absence of DAB signal in negative control sections (where the 84-1 antibody was excluded from the primary antibody incubation mixture). **b.** Tile scans of hippocampal sections stained with either a reference (Ref.) vimentin antibody or the 84-1 antibody. Boxes highlight the distinct reactivity patterns of 84-1 in pyramidal and granular neurons, while the Ref. vim showed a distinct signal only around the blood vessels. Scale bars: 20 µm in a.

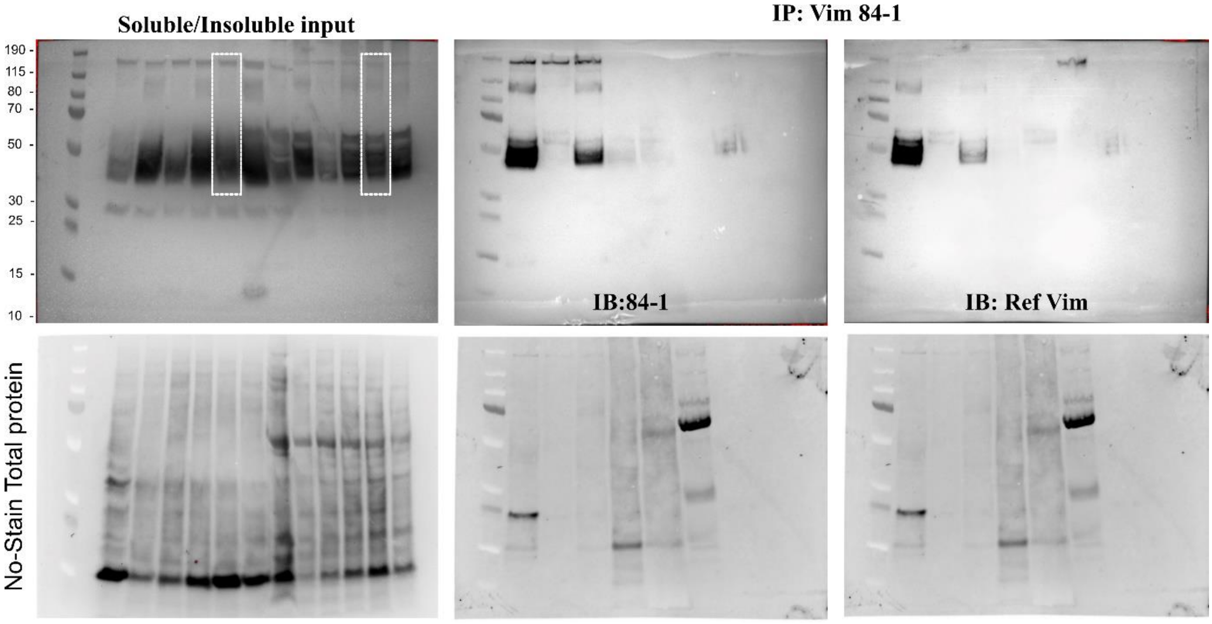

Supplementary Figure S6: Full uncropped membranes and Nostain total protein loading controls of membranes in Figure **3b.**

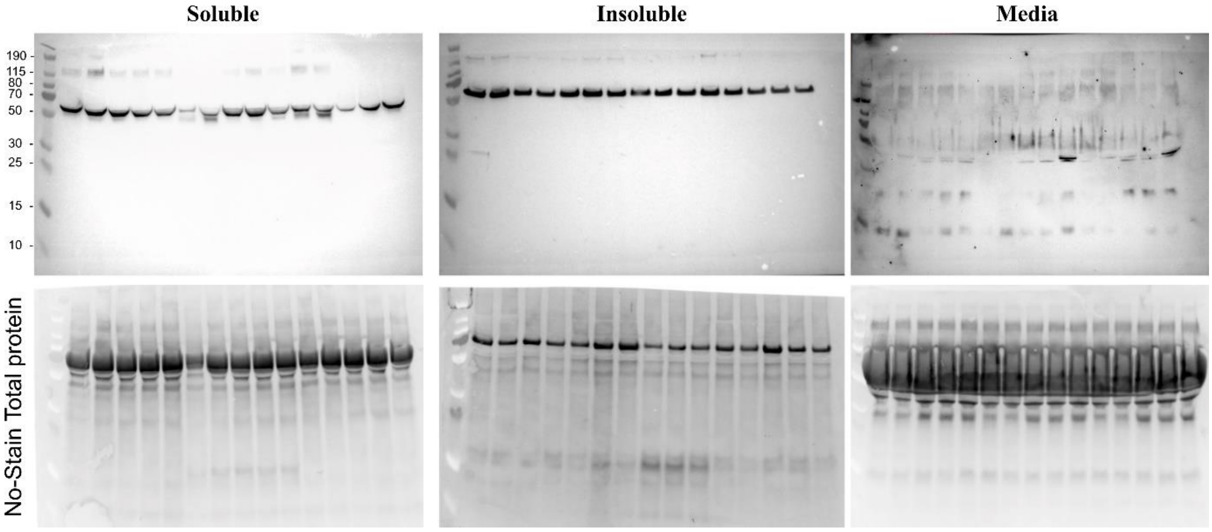

Supplementary Figure S7: Uncropped membranes (top row) and Nostain labelling of the same membranes (second row), showing the total protein for the western blot data in Figure **5c.**

Supplementary Table S6: List of reported modifications using the MSfragger tool in FragPipe

Index	Protein	Peptide	Localization Probability (S)	Localization Probability (Ins)	() Intensity	(Ins) Intensity
P08670_C328	sp|P08670|VIME_HUMAN	QSLTcEVDALK; QVQSLTcEVDALK; QVQSLTcEVDALKGTNESLER; RQVQSLTcEVDALK; RQVQSLTcEVDALKGTNESLER; SLTcEVDALK; SLTcEVDALKGTNESLER; TcEVDALKGTNESLER; cEVDALKGTNESLER	1	1	9.75E+09	5.25E+08
P08670_S214	sp|P08670|VIME_HUMAN	AsLARLDLERK	0	1	1.50E+08	1.87E+07
P08670_S261	sp|P08670|VIME_HUMAN	VsKPDLTAALR	0	1	0	4067375
P08670_S430	sp|P08670|VIME_HUMAN	PNFSSLNLRETNLDsLPLVDTHSK	0	1	0	372616.4
P08670_S72	sp|P08670|VIME_HUMAN	sSVPGVRLLQDSVDFSLADAINTEFK	1	0	2053150	0
P08670_T266	sp|P08670|VIME_HUMAN	VSKPDLtAALR	0	1	0	4067375
P08670_M14	sp|P08670|VIME_HUMAN	mFGGPGTASR;mFGGPGTASRPSSSR	1	1	5488510	2.08E+07
P08670_M154	sp|P08670|VIME_HUMAN	LGDLYEEEmR;LGDLYEEEmRELR	1	1	2.48E+08	7.05E+07
P08670_M183	sp|P08670|VIME_HUMAN	DNLAEDImR;VEVERDNLAEDImR	1	1	4.11E+08	4.80E+07
P08670_M193	sp|P08670|VIME_HUMAN	EKLQEEmLQR;EKLQEEmLQREEAENTLQSFR;LQEEmLQR;LQEEmLQREEAENTLQSFR	1	1	3.04E+08	6.77E+07
P08670_M344	sp|P08670|VIME_HUMAN	QmREmEENFAVEAANYQDTIGR	0	1	0	1784775
P08670_M347	sp|P08670|VIME_HUMAN	EmEENFAVEAANYQDTIGR;QmREmEENFAVEAANYQDTIGR	1	1	4.11E+08	1.30E+08
P08670_M372	sp|P08670|VIME_HUMAN	LQDEIQNmK;LQDEIQNmKEEMAR;LQDEIQNmKEEmAR	1	1	6.55E+08	1.27E+08
P08670_M376	sp|P08670|VIME_HUMAN	LQDEIQNMKEEmAR;LQDEIQNmKEEmAR	1	1	2.38E+08	6.64E+07
P08670_M391	sp|P08670|VIME_HUMAN	KmALDIEIATYR;mALDIEIATYR;mALDIEIATYRK	1	1	5.79E+08	1.06E+08
